# Experience‐Sensitive Effects on Temporal Profiles of Social Attention in Early Childhood

**DOI:** 10.1111/infa.70077

**Published:** 2026-03-21

**Authors:** Victoria St. Clair, Teresa Del Bianco, Emily J. H. Jones, Mairéad MacSweeney, Roberto Filippi, Peter Bright, Atsushi Senju, Evelyne Mercure

**Affiliations:** ^1^ Centre for Brain and Cognitive Development School of Psychological Sciences Birkbeck, University of London London UK; ^2^ School of Social Sciences and Professions London Metropolitan University London UK; ^3^ Institute of Cognitive Neuroscience University College London London UK; ^4^ Deafness, Cognition and Language Research Centre University College London London UK; ^5^ UCL Institute of Education London UK; ^6^ Division of Psychology Anglia Ruskin University Cambridge UK; ^7^ Research Center for Child Mental Development Hamamatsu University School of Medicine Hamamatsu Shizuoka Japan; ^8^ Department of Psychology Goldsmiths, University of London London UK

**Keywords:** bilingualism, child development, face processing, growth curve analysis, social attention

## Abstract

Bilinguals show differences in face processing compared to monolinguals, automatically orienting more rapidly to faces and dwelling longer on faces and mouths than monolinguals. However, it is difficult to identify specific visual strategies from average‐level data. This pre‐registered study uses growth curve analysis within trials to explore individual differences in monolingual and bilingual children's dynamic allocation of visual attention to static faces (“Face Pop‐Out”) and dynamic mouths (“50 Faces”). Participants were from Greater London in two age groups: 7‐ to 18‐month‐olds (*n* = 131) collected at the Birkbeck Babylab, and 18‐ to 34‐month‐olds (*n* = 745) whose data was publicly available from the Developing Human Connectome Project. Results show that children's attentional trajectories for viewing faces and mouths are sensitive to age and early language environment. Specifically, young bilinguals showed stronger systematic disengagement than monolinguals from faces and mouths after initial orientation. Older bilinguals prioritized the mouth more than monolinguals, driven by a steeper increase in mouth‐looking over stimulus time. Age‐dependent shifts in attentional allocation over stimulus time were evident within both age groups, particularly in static face viewing. In infants, younger children showed earlier re‐fixations to static faces than older children. In toddlers, attention to faces was more stable over stimulus time in older than younger children. Overall, results suggest that age and early exposure to two languages modulates the temporal structure of children's social attention from 7‐ to 34‐months of age.

## 1 Introduction

Simultaneous bilinguals (i.e., children who learn two languages from birth) face higher language learning demands and receive less exposure to each individual language compared to same‐age monolinguals (Hoff et al. 2012). Despite this, bilingual language development is remarkably similar to that of monolinguals. This might be because bilinguals develop adaptive strategies that support their dual‐language learning, such as heightened sensitivity to social cues in the environment (Birulés et al. 2018; Mercure et al. 2018; Mousley et al. 2023; Pons and Lewkowicz 2014; Pons et al. 2015). Many of these social cues are displayed by the human face as it conveys contextual information for language use (e.g., people's identities, Knappmeyer et al. 2003; O’Donnell and Bruce 2001; emotional states, Guarnera et al. 2018). Mouth movements provide a visual signal to support auditory speech processing (in adults: Król 2018; in children: Mercure et al. 2019). Differences in bilinguals' social attention compared to monolinguals may indicate increased attention to these signals and support young bilinguals' dual‐language learning. For example, research shows that bilingual infants maximize their exposure to human faces. Compared to monolinguals, bilinguals show faster speed of first saccades to static faces in an array (Mercure et al. 2018; Mousley et al. 2023), and when viewing videos of people speaking, higher overall looking time to mouths and stronger relative preference for mouths than eyes (Pons et al. 2015). These studies have also found relevant between‐subject variability, highlighting individual differences in social attention strategies amongst young bilinguals (Birulés et al. 2018). Crucially, such differences manifest in how attention is allocated over time and can be effectively analyzed through temporal profiles of social attention within trials. Growth curve analysis (“GCA”) provides a powerful method for capturing these individual differences, as it allows us to model fixation proportions over stimulus time. Using this technique, we can determine whether variability in gaze behavior over stimulus time stems from differences in language exposure or other individual‐level influences. Moreover, fixation probabilities over time offer a sensitive estimate of linguistic processing (Mirman et al. 2008), making this approach particularly well‐suited for investigating the interplay between language experience and social attention. The current study uses GCA to examine whether temporal profiles of attention to social stimuli over stimulus time are sensitive to early language experience in a large, heterogenous group of 7‐ to 34‐month‐old monolingual and bilingual children.

Roughly half the world's population uses more than one language and can broadly be considered “bilingual”, but there is substantial variability within this group in domains such as language exposure, fluency, and age of acquisition (Grosjean 2010). Bilinguals who learn two languages simultaneously from birth develop the early milestones of language development at roughly the same pace as monolinguals, but bilinguals do so in two languages. This requires adaptations in domains such as phoneme categorization, as native language pairs may have different overlap in their phonological composition (Bosch and Sebastián‐Gallés 1997), and differentiation of native languages from other languages and from each other (Byers‐Heinlein et al. 2010; Werker 2012). It might be that bilinguals develop adaptive strategies in the domain of visual attention to support their early dual‐language learning. For example, bilinguals may orient their attention to faces faster than monolinguals to make better use of visual articulation cues if the face were to begin to speak.

One way to measure how interested an infant is in faces is to measure their orientation to a face in an array of other non‐social objects (Gliga et al. 2009; Gluckman and Johnson 2013). Whilst infants of all language groups tend to direct their attention faster to and spend longer looking at faces compared to other non‐face areas, this “face pop out” phenomenon is influenced by early language experience (Mercure et al. 2018; Mousley et al. 2023). Compared to monolinguals, bilinguals from 7‐ to 18‐months‐old make more rapid first saccades toward pictures of faces presented with distractors (i.e., a face‐popout task, Gliga et al. 2009; Mercure et al. 2018; Mousley et al. 2023), and look more frequently to the face compared to distractors than monolinguals at 7 and 10 months (Mercure et al. 2018), but not at 15 and 18 months (Mousley et al. 2023). Importantly, social attention to faces and the face's internal features shifts along a timeline corresponding to milestones of language acquisition. For example, monolingual children's proportion of looking to eyes of talking faces peaks at 4‐month‐olds and is surpassed by more mouth looking by 10‐month‐olds (Lewkowicz and Hansen‐Tift 2012). Allocating more attention to the mouth than the eyes by 10 months corresponds with a developmental period of intense phonological learning (Werker 2018) and the emergence of canonical babbling (e.g., Chandrasekaran et al. 2009; Lewkowicz and Kraebel 2004).

Crucially, shifts in mouth looking behavior are sensitive to early language experiences (Birulés et al. 2018; Mercure et al. 2018, 2022; Pons and Lewkowicz 2014; Pons et al. 2015; Teinonen et al. 2008), with bilinguals looking longer than monolinguals to talking mouths both at earlier and later ages (e.g., 4 and 12 months, Pons et al. 2015). Whilst watching speakers, monolinguals showed a preference for the eyes at 4 and 8 months, whilst bilinguals looked equally to eyes and mouths at 4 months and more to the mouths than eyes at 8 months (Pons et al. 2015). Other studies using similar audiovisual speaking stimuli suggest no difference in mouth looking between bilinguals and monolinguals in early infancy and childhood (Mercure et al. 2019; Morin‐Lessard et al. 2019) — potentially because of within‐group variability of bilinguals' experiences, such as degree of similarity between their two native languages (Birulés et al. 2018).

Building on this, the current study leverages a large, diverse sample drawn from two age groups, encompassing bilingual children exposed to various language combinations, including close (e.g., English and Dutch) and distant language pairs (e.g., English and Arabic). Expanding previous studies that relied on averaged measures of looking behavior across time, we examine within‐trial temporal profiles of social attention of bilingual and monolingual children using both dynamic and static stimuli. This is important because previous research often reports differences in averaged measures (e.g., total looking time across many trials) and suggests differences between monolinguals and bilinguals might relate to different visual strategies between the groups. However, probing group differences in visual strategies requires measuring them directly. Here, we modeled directly trajectories of visual attention over stimulus time to examine whether children's language backgrounds or ages related to different visual strategies. We examined two age groups: infants aged 7–18 months and toddlers aged 18–34 months. Previous research suggests that over this period, early language experience modulates social attention in aggregate‐level constructs (e.g., proportion of total looking time to talking mouths in the first year, Pons et al. 2015; speed of first look to static faces in the second year, Mousley et al. 2023). We therefore sought to investigate differences between monolinguals and bilinguals' attentional trajectories over stimulus time which might underlie trial‐level differences. In line with existing literature, we predicted that bilingual participants would show (1) in a static face‐pop out task, early onset of fixations to faces compared to distractors compared to monolinguals (Mousley et al. 2023) and, (2) when watching dynamic videos, higher proportional looking time to mouths than monolinguals (Pons et al. 2015). These hypotheses were pre‐registered on the Open Science Framework: https://osf.io/8j5cg.

We used data from two large studies to test the effect of early language experience on temporal profiles of social attention. A sample of 7‐ to 18‐month‐old infants (*n* = 131) included in‐depth information about children's language backgrounds that allowed for a categorical distinction between monolinguals and bilinguals, alongside within‐group factors for each bilingual's experience, such as degree of bilingualism and language mixing scores (see Method for details). We expected degree of bilingualism and language mixing scores to be positively related to speed of orientation to faces and overall looking to mouths. A sample of 18‐ to 34‐month‐olds (*n* = 745) included toddlers with either an English‐speaking monolingual mother or bilingual mothers who spoke English and one of 83 different non‐English languages.

## 2 Method

### 2.1 Power Calculations

Power calculations were conducted to estimate the sample sizes required to detect bilingual effects on each task using traditional statistical methods (e.g., ANOVA) with 95% confidence (see Supporting Information S1 for more detail). Effect size estimates were used from previous literature reporting bilingual effects on similar tasks with same‐aged children. The calculations indicated a necessary sample of *N* = 99 for Face Pop‐Out and *N* = 52 for the Dynamic Video task. We therefore had sufficient power in both infant (*n* = 131) and toddler (*n* = 745) age groups to detect effects of interest for both tasks.

### 2.2 Participants

**FIGURE 1:**
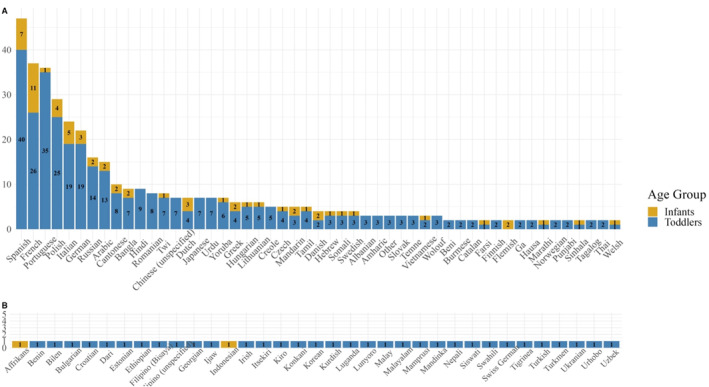
Non‐English languages of bilingual infants and toddlers' bilingual mothers. 7‐ to 18‐month‐old bilingual infants (*n* = 131) non‐English languages represented in yellow. 18 to 34‐month‐old (*n* = 745) toddlers' bilingual mothers' non‐English languages represented in blue. Some children were exposed to more than one non‐English language and therefore appear more than once on this list. Languages listed as written by parents. (A) Shows languages used by more than one participant. (B) Shows languages used by only one participant.

A total of *N* = 899 children between the ages of 7 and 18 months participated across two different age groups. A total of *n* = 23 were excluded for failure to complete eye‐tracking tasks, resulting in a final sample of *N* = 876 datasets for analysis (*n* = 368 bilingual, see Figure 1). Data was collected by two different research teams located in London. Infants (7–18 months) were recruited as part of bilingualism‐specific research projects at Birkbeck, University of London, and data from this sample have been written‐up elsewhere using traditional, aggregated statistical techniques (Mercure et al. 2018; Mousley et al. 2023). Toddlers (18–34 months) participated in the Developing Human Connectome Project at King's College London (Edwards et al. 2022). More detail about each age group below and in Supporting Information S1: Figures S1 and S2. The present study was conducted according to guidelines laid out in the Declaration of Helsinki, with written informed consent obtained from a parent or guardian for each child before any assessment or data collection. All procedures involving human subjects in this study were approved by UCL and Birkbeck ethics committees (for infant data) and United Kingdom National Research Ethics Authority (for toddler data).

### 2.3 Infants (7‐ to 18‐Month‐Olds)

Data from infants (*n* = 131) aged 7‐ to 18‐months were collected across two studies at the Center for Brain and Cognitive Development at Birkbeck, University of London. Infants were recruited as monolinguals if they were exposed only to English, and as bilinguals if parents reported their children were regularly exposed to a non‐English language. Language background questionnaires with bilingual infants' parents were conducted to calculate their percentage of exposure to each language and their parents' tendency to mix languages when they speak to their children. See Table 1 for sample background characteristics and Figure 1 for list of non‐English languages.

**TABLE 1:** Sample background characteristics.

	Gender	Age (days)	
	(Number girls)	*M* (SD)	Min–Max
Infants (7–18 months)			
Monolinguals (*n* = 72)	*n* = 32	420.22 (127.93)	222–577
Bilinguals (*n* = 59)	*n* = 24	395.73 (127.38)	221–557
Toddlers (18–34 months)			
Monolingual mother (*n* = 436)	*n* = 210	582.02 (75.29)	457–1037
Bilingual mother (*n* = 309)	*n* = 139	571.92 (63.32)	520–1039
Total			
Monolingual or with a monolingual mother (*n* = 508)	*n* = 242	560.20 (99.31)	222–1037
Bilingual or with bilingual mother (*n* = 368)	*n* = 163	540.17 (104.04)	221–1039

*Note:* All data here lists parental report of their children's gender, though data from toddlers appear to report biological sex (i.e., as male vs. female). Across all children, gender/sex were only ever reported along the binary of boy/girl and male/female. We report this information here as “gender” for continuity, as we assume this was the intention for both age groups. Toddlers were classified according to mothers' language use (i.e., monolingual English‐speakers or bilingual users of English and a non‐English language).

#### 2.3.1 Language Exposure Questionnaire (“LEQ”)

The LEQ, designed by Bosch and Sebastián‐Gallés (1997) and used widely in developmental research on bilingualism (Carbajal and Peperkamp 2020; DeAnda et al. 2016; Kalashnikova et al. 2020; Potter et al. 2018; Singh 2018; Singh and Tan 2020), takes between 10 and 15 min to administer. Parents are walked through an infant's typical day for each day of the week and asked which languages are spoken directly to their child at different times (i.e., home, nursery, during typical weekday and weekend activities). The number of hours an infant hears their two native languages is calculated and a percentage score of exposure to each language is produced. Each bilingual infant's percentage of language exposure was used to derive a “degree of bilingualism” calculated as the percentage of exposure to the less dominant language divided by percentage of exposure to the more dominant language (e.g., 40% English/60% Russian). Thus, a score near 1.0 indicates a 50/50 split, whilst a score closer to 0.25 indicates a 20/80 split. Developmental research with bilingual children, when using categorical group inclusion, often defines bilingualism as a function of an infant's percentage of exposure to two languages (e.g., 20% English, 80% Spanish). However, the threshold necessary to be considered a bilingual child varies widely across studies (Byers‐Heinlein 2015). We were interested in whether previous findings about differences in bilinguals generalize to a heterogenous sample and therefore did not exclude children based on their degree of bilingualism. In infants, bilinguals on average reported a degree of bilingualism of *M* = 0.40 (SD = 0.18, min = 0.06, max = 0.96). Bilinguals' average exposure to English was *M* = 53.35% (SD = 0.24, min = 6.65%, max = 93.35%). Bilinguals' average exposure to their non‐English language was *M* = 46.74% (SD = 0.24, min = 0.05%, max = 93.35%).

#### 2.3.2 Language Mixing Scales

Bilingual parents were also asked to complete a three‐ to five‐minute language mixing scale. Parents were asked to rate, on a scale of zero (Never) to six (Always), how often they mixed their language use. Items included behaviors such as borrowing words from another language and code switching between two languages in the same sentence. This measure has been used previously in research on bilingualism (Byers‐Heinlein 2013; Byers‐Heinlein et al. 2020; Tsui et al. 2020). Bilingual parents reported, on average, a language mixing score of *M* = 12.63 (SD = 6.56, min = 1, max = 30) out of a total possible 36 points on the language mixing scale.

### 2.4 Toddlers (18‐ to 34‐Month‐Olds)

Data from toddlers (*n* = 745) was accessed from the publicly available Developing Human Connectome Project, which is a longitudinal examination of neurodevelopment in children from the Greater London area that collects eye‐tracking data from 18‐ to 34‐month‐olds (details and link to data found in Edwards et al. 2022). The primary research questions of the original project are not related to language background and extensive language background interviews were not conducted. However, all toddlers were being raised in the Greater London area of the United Kingdom and therefore had some experience with English, but many were also likely from diverse early language backgrounds. The dataset lists the first language of each participant's mother and father, which was used to categorize toddlers' mothers as either monolingual English‐speakers or bilingual users of English and a non‐English language (see Figure 1). The nature of the language group variable was therefore different in the sample of toddlers than in infants: bilingual infants were categorized according to direct quantification of early language exposure, whilst toddlers were grouped according to mothers' language status as a proxy for possible bilingualism of the toddler. In toddlers, there was one instance where a mothers' first language was unknown, but the participant was noted as only hearing English at home, so this child was classified as having a monolingual mother. In one instance, a toddler's mother was listed as a native English speaker but also had a non‐English first language listed. This toddler was classified as having a bilingual mother. See Figure 1 for full list of bilingual mothers' non‐English languages.

All analyses with toddlers were first run as a comparison between those whose mothers were monolingual versus bilingual; however, it was later considered that calculating a numeric score for language group may more accurately reflect the heterogeneity of the data. In an exploratory step, all models in toddlers were run with language experience as a numeric predictor, such that toddlers with two monolingual parents were assigned a score = 0, one native non‐English parent (either mother or father) as = 1, and two native non‐English parents = 2. Results for numeric group definition are included in Supporting Information S1.

### 2.5 Stimuli and Procedure

**FIGURE 2:**
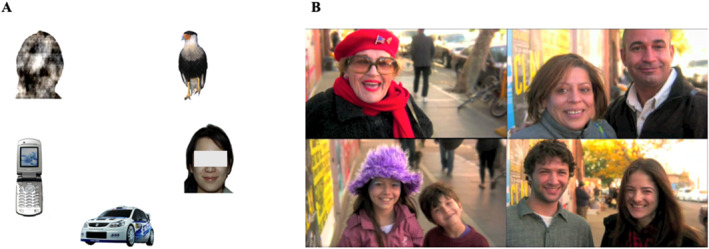
Depiction of face Pop‐Out and dynamic video tasks. (A) Face Pop‐Out slide containing five static areas of interest, including a face of different races and genders, smiling softly and making direct eye‐contact. (B) Dynamic Video still frames. Participants were recorded answering the question: “Where would you wish to wake up tomorrow?”

Eye‐tracking data were collected using either a Tobii T120 (*n =* 46) or a TX300 (*n* = 85) eye‐tracker (Tobii, Danderyd, Sweden). TX300 data were acquired at a maximum sampling rate of 300 Hz. For the T120 data, *n* = 25 were collected at 60 Hz and *n* = 21 at 120 Hz. Analyses did not reveal differences in left or right eye accuracy between participants collected at 60 versus 120 Hz (left eye: *t*(42.07) = 1.75, *p* = 0.087; right eye: *t*(42.38) = 1.89, *p* = 0.065). Infant data were collected either in TobiiStudio (*n* = 46) or using a custom‐written stimulus presentation framework (*n* = 85) running in MATLAB using Psychtoolbox 3 (Brainard 1997; Kleiner et al. 2007) and the GStreamer library for video decoding (gstreamer.freedesktop.org). Raw gaze was recorded and processed with Tobii Gaze Analytics SDK 3.0. Eye‐tracking data were collected while participants passively viewed stimuli in the following two experiments:

1. **Face Pop‐Out:** Trials presented static arrays containing one face of different genders and races, a scrambled face, a car, a bird, and a mobile phone for approximately 10 s (Gliga et al. 2009; Mercure et al. 2018; Mousley et al. 2023). Placement of areas were randomized in each trial (see Figure 2A for an example of one trial, anonymized stimuli slides found on OSF: https://osf.io/8j5cg).
2. **Dynamic Video:** A 40‐s excerpt from “50 People, One Question Brooklyn”, containing interviews with 13 people, balanced by gender, on the street answering the question “Where would you wish to wake up tomorrow?” whilst relaxing piano music plays in the background (Võ et al. 2012; see Figure 2B). Faces were talking for approximately 60.25% of the total trial time, during which their spoken language was audibly presented above the quiet background music as is standard in previous research (Del Bianco et al. 2022).

All participants sat centrally approximately 60 cm away from the screen. Five‐point calibration was performed before stimulus presentation began. For both age groups, static and dynamic face task blocks were interleaved with other eye‐tracking tasks to maintain children's attention. A central fixation point containing a neutral object (e.g., schematic ball, cartoon house) occurred between each trial. The battery progression was gaze contingent such that the participant was required to fixate on the central point before the next trial was presented. All children were presented with eight static Face Pop‐Out trials and saw 40‐s of the Dynamic Video.

### 2.6 Data Processing

Areas of interest (“AOIs”) were manually drawn on the static face areas in the Face Pop‐Out task and on each frame of the Dynamic Video task. Gaze samples were collected at 60, 120, or 300 estimates per second as determined by the sampling rate of the eye‐tracker (see above). Each gaze sample was scored according to whether the child's gaze fell within any of the AOIs. The Face Pop‐Out task was presented as eight discrete trials. The Dynamic Video task was coded in ELAN video coding software to mark the onset of appearance of each individual face over the 40‐s total duration of the video (ELAN 2019). These timestamps were used to segment the gaze data into 13 scenes in which faces were present. For both tasks, gaze samples were grouped into half‐second time bins, and proportional looking time (“PLT”) was calculated for each group. PLT was defined as the number of gaze samples within the area of interest divided by the total number of valid gaze samples, with exclusion of time bins with > 75% missing data. Due to a technical error during eye‐tracking acquisition for infants' Face Pop‐Out task, one sample (second 10.807952) was corrupted and therefore excluded for all infants.

#### 2.6.1 Quality Checks

Data were checked to ensure no systematic differences in quality across age groups. There were no participants in either age group or either task where all scenes/trials were excluded. Sample‐level missing gaze data were not available for the Face Pop‐Out task for *n* = 46 infant participants collected in TobiiStudio. For these participants, those who looked at the entire slide for less than one second were excluded (as in Mercure et al. 2018). In neither infants nor toddlers did the proportion of missing data for Face Pop‐Out slides (see Supporting Information S1: Tables S2 and S3) or the Dynamic Video (see Supporting Information S1: Tables S4 and S5) differ by age, gender, or language group.

### 2.7 Statistical Analysis

Growth curve analysis (“GCA”) was applied to examine differences in attention to static faces (Hypothesis 1: Face Pop‐Out) and dynamic mouths (Hypothesis 2: Dynamic Video) between monolingual and bilingual children (Del Bianco et al. 2022; Mirman et al. 2008). GCA models the change in fixation proportions over stimulus time by incorporating orthogonal polynomials of time as fixed effects, capturing nonlinear trends in gaze behavior. This allows us to assess how attention unfolds dynamically and how it interacts with key predictors such as language background. For a visual representation of temporal trends as measured by polynomials, see Supporting Information S1: Figure S2 (Del Bianco et al. 2021).

To account for individual variability, we implemented GCA within a mixed‐effects framework, including random intercepts per participant (see Supporting Information S1: Table S6). Age and gender, both shown in research with older children to affect temporal profiles of attention (Del Bianco et al. 2022), were tested as covariates before building models of interest, and results of covariate Likelihood Ratio Tests can be found in Supporting Information S1 (see Tables S7 and S18 for infants, S13 and S25 for toddlers). Significant covariates were then included in the models of interest, which included polynomial components and their interaction with children's language group (see Supporting Information S1: Table S6). The models were fit using maximum likelihood estimation and assessed for goodness of fit. Temporal trends across all children are reported from main effect models (model construction 4 in Supporting Information S1: Table S6 for Face Pop‐Out and 3 for Dynamic Video, see Supporting Information S1: Tables S8 and S19 for infants, S14, and S26 for toddlers). Post‐hoc analyses were conducted to explore the nature of any significant effects observed.

Original scripts written by Del Bianco et al. (2022) can be found at https://github.com/vmousley/GCA and were adapted for the samples in this study. Following Del Bianco et al. (2022), orthogonal polynomials of stimulus presentation time were computed up to degree three for Face Pop‐Out and degree two for the Dynamic Video. Degree three polynomials account for three shifts in attentional focus (linear, quadratic, and cubic components), while degree two captures two shifts (linear and quadratic components). This approach allows for a fine‐grained examination of how gaze behavior evolves throughout the trial and how bilingual and monolingual children differ in their social attention patterns.

Language differences in temporal attention profiles were modeled using a stepwise addition procedure with Likelihood Ratio Tests (Lai et al. 2013; Del Bianco et al. 2022; see Supporting Information S1: Table S6 for detail). For each task, group differences were first examined in infants aged 7–18 months, where language background interviews allowed for categorization of children as either monolingual or bilingual. When significant group differences emerged, within‐group variability was explored based on degree of bilingualism—as measured by the LEQ (Bosch and Sebastián‐Gallés 1997), the ratio of exposure to the less dominant versus more dominant language, for example, 20% English/80% Russian = 0.25—and the degree to which parents reported mixing their two languages when speaking with their children. Within‐group results are reported in the Supporting Information S1 (see Tables S10 and S24). Group differences were then tested in toddlers aged 18–34 months according to the pre‐registered plan. A summary of results is provided in Table 2.

**TABLE 2:** Summary of primary results.

	Average profile (intercept)			Dynamic profile (polynomial)	
	Age	Gender	Language group	Age	Language group
Face pop‐out: Looking to faces (hypothesis 1)					
Infants: 7–18 months	Overall more face‐looking in younger > older infants (age, *p* = 0.011)	n.s.	n.s.	Early return to face stronger in younger > older infants (quadratic x age, *p* < 0.001) Later return to face stronger in older > younger infants (cubic x age, *p* < 0.001).	Net decline in face‐looking from start to end steeper in bilinguals > monolinguals (slope × language group, *p* = 0.016)
Toddlers: 18–34 months	Overall more face‐looking in younger > older toddlers (age, *p* = 0.040)	n.s.	n.s.	Initial decline in face‐looking after early peak stronger in younger > older toddlers (quadratic x age, *p* = 0.007) Later return to face stronger in younger > older toddlers (cubic x age, *p* = 0.016) Net decline in face‐looking from start to end stronger in younger > older toddlers (slope × age, *p* < 0.001)	n.s.
Dynamic video: Looking to mouths (hypothesis 2)					
Infants: 7–18 months	Overall more mouth‐looking in older > younger infants (age, *p* < 0.001)	Overall more mouth‐looking in girls > boys (gender, *p* = 0.025)	n.s.	n.s.	Net decline in mouth‐looking from start to end steeper bilinguals > monolinguals (slope × language group, *p* = 0.029)
Toddlers: 18–34 months	n.s.	n.s.	Overall more mouth‐looking in those with bilingual > monolingual mothers (language group, *p* < 0.001)	n.s.	Increase in mouth‐looking from start to end stronger in those with bilingual > monolingual mothers (slope × language group, *p* = 0.019) Trend of stronger late peak in mouth‐looking in those with bilingual > monolingual mothers (quadratic × language group, *p* = 0.085)

*Note:* n.s. indicates non‐significant effects. Language group in infant sample calculated according to direct measurement of the infants' early language exposure to English (monolingual) or English and another non‐English language (bilingual). In toddlers, language group was measured in terms of mothers' first language (i.e., monolingual if English, bilingual if non‐English).

Finally, four exploratory analyses were conducted. Age effects were tested on a collapsed sample of infants and toddlers (see Supporting Information S1: Tables S29–S32) for both tasks. For the Dynamic Video, within both infants and toddlers separately, age and language group effects were tested on looking to the eye and overall face areas of interest (see Supporting Information S1: Tables S33–S40). Bilingual infants' percentage of exposure to English, the language used by the Dynamic Video stimuli, was also explored in relation to mouth‐looking (see Supporting Information S1: Table S24). Finally, distance between language pairs was explored in relation to looking to face (see Supporting Information S1: Tables S41–S43) and mouths (see Supporting Information S1: Tables S41, S44, and S45).

## 3 Results

### 3.1 Face Pop‐Out: Looking to Faces

#### 3.1.1 Infants (7‐ to 18‐Month‐Olds)

##### 3.1.1.1 Average Profiles

There was a significant effect of age on face looking such that younger infants looked more to the face than older infants (Age, Coef = −2.48 × 10^−4^ [−3.57 × 10^−4^ ∼ −9.63 × 10^−6^], SE = 9.61 × 10^−5^, *p* = 0.011, see Supporting Information S1: Tables S7–S12). There were no differences in monolingual and bilingual face looking at an aggregate level (*p* = 0.197, see Supporting Information S1: Tables S9 and S12).

##### 3.1.1.2 Temporal Profiles

**FIGURE 3:**
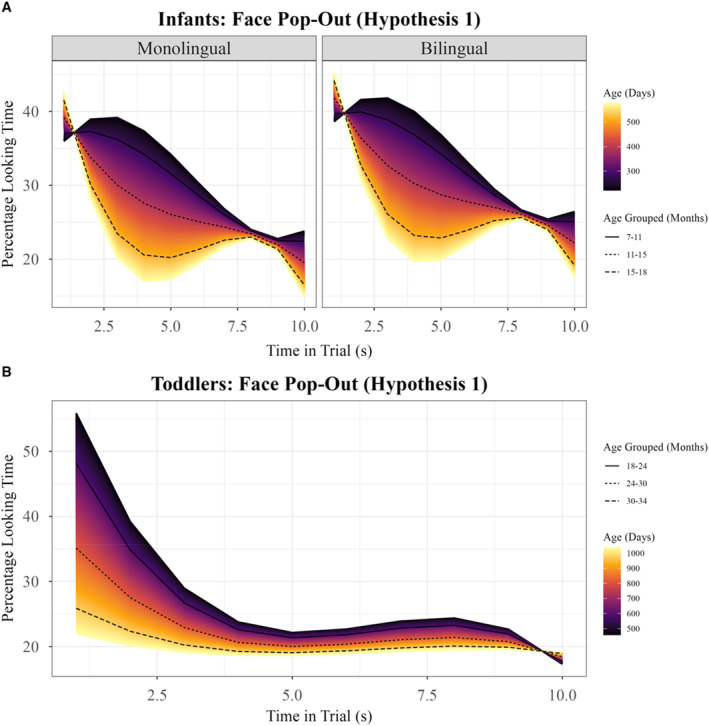
Growth curve models for face‐looking—Both age groups. Representation of growth curve models for attention trajectories to faces in Face Pop‐Out in both age groups. Percentage looking time calculated as percentage of samples falling within face area out of total number of valid samples averaged per 0.5‐s time bin. (A) Representation of best fit model for infants (see Supporting Information S1: Table S12). Color represents interaction between age as a continuous variable and temporal profiles. Black lines represent average estimate for age grouped by months. Left pane shows fit in monolinguals and right pane in bilinguals. (B) Representation of best fit model for toddlers (see Supporting Information S1: Table S17). Toddlers with both monolingual and bilingual mothers are grouped as models revealed no effect of language group. Color represents interaction between age as a continuous variable and temporal profiles. Black lines represent average estimate for age grouped by months.

The model indicated that, in both bilingual and monolingual groups, infants oriented their attention to the face first before shifting their attention away to another area in the array (Quadratic, Coef = 0.06 [0.03–0.08], SE = 0.01, *p* < 0.001, see Supporting Information S1: Table S8). The rate of disengagement from the face across the trial differed between groups, with the overall net decline in face‐looking significantly steeper in bilinguals than monolinguals (Slope × Language Group interaction, Coef = −0.07 [−0.13 ∼ −0.01], SE = 0.03, *p* = 0.016, see Supporting Information S1: Table S12). Bilinguals' steep slope indicated that they were quicker to stop focusing on the face compared to the more gradual decline over stimulus time observed in monolinguals (see Figure 3A, see Supporting Information S1: Table S12). A later return of attention to the face was observed in both groups (Cubic, Coef = −0.05 [−0.08 ∼ −0.02], SE = 0.01, *p* < 0.001, see Supporting Information S1: Table S8), demonstrating a second shift in focus as children re‐engaged with the face.

Age significantly influenced both average attention and temporal profiles, improving model fit when included as an interaction term with third‐degree polynomials (see Supporting Information S1: Tables S11 and S12). Age modulated the timing of attention shifts, with younger infants showing a stronger early return to faces (Quadratic × Age interaction, Coef = 4.44 × 10^−4^ [2.24 × 10^−4^ ∼ 6.22 × 10^−4^], SE = 1.02 × 10^−4^, *p* < 0.001, see Supporting Information S1: Table S12), whilst older infants' later peak was later in stimulus time (Cubic × Age interaction, Coef = −5.99 × 10^−4^ [−8.05 × 10^−4^ ∼ −3.83 × 10^−4^], SE = 1.09 × 10^−4^, *p* < 0.001, see Supporting Information S1: Table S12). The overall decline in attention to the face from the start to the end of the trial—stronger in bilinguals than monolinguals, but evident across all infants—was not affected by age (Slope × Age interaction, *p* = 0.588, see Supporting Information S1: Table S12). These findings suggest that as all children grow, their attentional re‐engagement with faces shifts to later points in stimulus time.

#### 3.1.2 Toddlers (18‐ to 34‐Month‐Olds)

##### 3.1.2.1 Average Profiles

There was a significant effect of age such that younger toddlers looked more to faces overall than older toddlers (Age, Coef = −1.47 × 10^−4^ [−2.88 × 10^−4^ ∼ −6.88 × 10^−6^], SE = 7.17 × 10^−5^, *p* = 0.040, see Supporting Information S1: Tables S13, S15–S17, see Figure 3B). There were no language group differences in looking to faces at an aggregate level (*p* = 0.931, see Supporting Information S1: Table S15).

##### 3.1.2.2 Temporal Profiles

All toddlers showed high initial attention to the face followed by a relative decrease (Quadratic, Coef = 0.13 [0.12–0.15], SE = 0.01, *p* < 0.001, see Supporting Information S1: Table S14) and a later peak in returning to the face (Cubic, Coef = −0.11 [−0.12 ∼ −0.10], SE = 0.01, *p* < 0.001, see Supporting Information S1: Table S14). Across the trial, there was an overall net decline in face‐looking from start to finish (Slope, Coef = −0.21 [−0.23–7.38 × 10^−5^], SE = 0.01, *p* < 0.001, see Supporting Information S1: Table S14). Younger toddlers showed a stronger initial decline in face looking than older toddlers after the early peak (Quadratic × Age interaction, Coef = −2.44 × 10^−4^ [−4.20 × 10^−4^ ∼ −6.76 × 10^−5^], SE = 8.98 × 10^−5^, *p* = 0.007, see Supporting Information S1: Table S17), stronger later return to the face (Cubic × Age interaction, Coef = 2.02 × 10^−4^ [3.87 × 10^−5^ ∼ 3.65 × 10^−4^], SE = 8.32 × 10^−5^, *p* = 0.016, Supporting Information S1: Table S17), and more overall disengagement from start to end than older toddlers (Slope × Age interaction, Coef = 4.40 × 10^−4^, [2.42 × 10^−4^ ∼ 6.38 × 10^−4^], SE = 1.01 × 10^−4^, *p* < 0.001; Supporting Information S1: Table S17). No significant differences were found between those with a monolingual versus a bilingual mother (see Supporting Information S1: Table S15).

### 3.2 Dynamic Video: Looking to Mouths

#### 3.2.1 Infants (7‐ to 18‐Month‐Olds)

##### 3.2.1.1 Average Profiles

Age and gender significantly influenced average mouth looking, with older girls looking longer to mouths overall than younger boys (Age, Coef = 4.32 × 10^−4^ [2.53 × 10^−4^ ∼ 6.10 × 10^−4^], SE = 9.11 × 10^−5^, *p* < 0.001; Gender, Coef = −0.05 [−0.10 ∼ −0.01], SE = 0.02, *p* = 0.025, see Supporting Information S1: Tables S18–S21). There were no differences in mouth looking between language groups at an aggregate level (*p* = 0.134; see Supporting Information S1: Table S23).

##### 3.2.1.2 Temporal Profiles

Monolinguals' attention to the mouths was not significantly predicted by any of the polynomial predictors, suggesting that monolinguals' mouth‐looking remained stable and that they did not systematically engage with the mouth in a way that followed the trial's temporal dynamics (see Supporting Information S1: Tables S20 and S23). There was an interaction between the timing of attentional shifts and language group (see Supporting Information S1: Table S20), driven by bilinguals' general bias for the mouth that gradually decreased with stimulus time as they redirected their attention to other areas (Slope × Language Group interaction, Coef = −0.03 [−0.06 ∼ −3.58 × 10^−3^], SE = 0.01, *p* = 0.029, see Supporting Information S1: Table S23).

#### 3.2.2 Toddlers (18‐ to 34‐Month‐Olds)

##### 3.2.2.1 Average Profiles

Toddlers with a bilingual mother showed significantly more mouth‐looking overall than those with a monolingual mother (Language Group, Coef = 0.04 [0.02 ∼ 0.07], SE = 0.01, *p* < 0.001, see Supporting Information S1: Tables S27 and S28).

##### 3.2.2.2 Temporal Profiles

**FIGURE 4:**
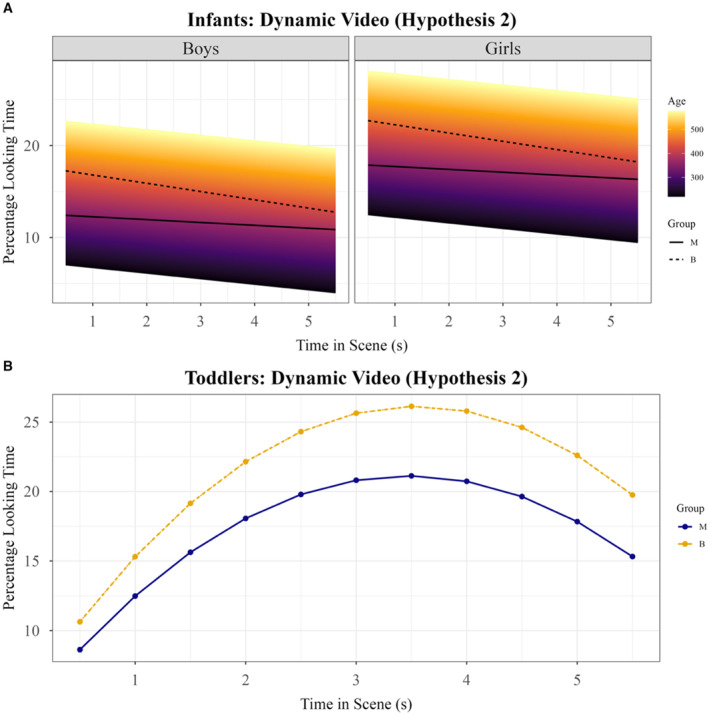
Growth curve models for mouth looking—Both age groups. Representation of growth curve models for attention trajectories to mouths in the Dynamic Video task in both age groups. Percentage looking time calculated as percentage of samples falling within face area out of total number of valid samples averaged per 0.5‐s time bin. (A) Representation of best fit model for infants (see Supporting Information S1: Table S23). Color represents main effect of age. Black lines represent average for each language group. Left pane shows fit in boys and right pane in girls. (B) Representation of best fit model for toddlers (see Supporting Information S1: Table S28). Orange line represents estimates for toddlers with a bilingual mother and blue for those with a monolingual mother.

The model indicated an overall increase in attention to mouths from the beginning to the end of the trial for all toddlers (Slope, Coef = 0.08 [0.07–0.09], SE = 0.01, *p* < 0.001, see Supporting Information S1: Table S26), suggesting a gradual engagement with the mouth over stimulus time. This increase was significantly steeper in those with a bilingual mother compared to those with a monolingual mother (Slope × Language Group interaction, Coef = 0.03 [0.00–0.05], SE = 0.01, *p* = 0.019, see Supporting Information S1: Table S28 and Figure 4B), indicating that toddlers with a bilingual mother were more likely than those with a monolingual mother to shift attention dynamically toward the mouth throughout the trial. Additionally, a late peak in mouth‐looking was observed for all toddlers (Quadratic, Coef = −0.11 [−0.12 ∼ −0.10], SE = 0.01, *p* < 0.001, see Supporting Information S1: Table S26), reflecting a non‐linear pattern where gaze initially increased, then briefly decreased, before returning to the mouth later in the trial. There was a trend suggesting that this late re‐engagement was stronger in those with a bilingual than a monolingual mother (Quadratic x Language Group interaction, Coef = −0.02 [−0.04 ∼ −0.00], SE = 0.01, *p* = 0.085; see Supporting Information S1: Table S28), though this effect did not reach significance. This pattern suggests that toddlers with a bilingual mother engage in dynamic allocation of attention, with a steeper increase in mouth‐looking over stimulus time and possibly in later re‐engagement compared to those with a monolingual mother.

## 4 Discussion

This study examined the temporal dynamics of face‐looking in a large, language‐diverse group of children. The results reveal structured patterns of attentional shifts influenced both by language experience and age. Broadly, effects of bilingualism on attentional trajectories were characterized by stronger systematic disengagement with social stimuli over trials compared to monolinguals at 7–18 months, and stronger increasing allocation to mouths over stimulus time in 18‐ to 34‐month‐olds with bilingual mothers compared to those with monolingual mothers. 7‐ to 18‐month‐olds’ trajectories of attention to static faces, but not to dynamic mouths, over stimulus time also showed a clear developmental shift with age, where older infants re‐fixated on the face later into stimulus time than younger infants.

### 4.1 Face Pop‐Out and Face Looking

The Face Pop‐Out task was designed to assess children's orienting to faces compared to non‐faces in a static array (Gliga et al. 2009). Across both language and age groups (7‐ to 18‐month‐old infants and 18‐ to 34‐month‐old toddlers), children initially oriented to faces before shifting attention away and later returning, as captured by the quadratic and cubic patterns (see Supporting Information S1: Table S8 for infants and S14 for toddlers). Bilingual infants showed a steeper negative slope of face‐looking over stimulus time compared to monolinguals, characterized by an initial strong preference for the face before disengaging and freeing up their attention to look elsewhere (see Figure 3A). In contrast, monolingual infants' trajectory of looking to faces was flatter and more similar at the start and end of the trial than bilingual infants'. Bilingual infants' faster rate of change compared to monolinguals may reflect greater attentional flexibility, a pattern that aligns with the theory that bilinguals' varied language environments might drive a stronger earlier orienting response and similar later engagement with faces compared to monolinguals. Within the infant bilingual group, neither patterns of children's language exposure nor parental language mixing predicted individual differences in attentional shifts away from faces (see Supporting Information S1: Table S10). While bilingual disengagement from the face was not directly explained by variation in bilingual experience within the infant sample, the bilingual effect on gaze shifts may reflect broader attentional adaptations rather than differences in individual exposure. This pattern suggests that even little but regular exposure to two languages could lead to adaptations in attentional profiles, and that such adaptations might not be “dose‐dependent.”

Stronger visual disengagement in bilinguals than monolinguals is consistent with D'Souza et al. (2020), who found that 7‐ to 9‐month‐old bilinguals more readily disengaged from one (non‐social) stimulus to another and more often switched their attention between familiar and novel stimuli than same‐aged monolinguals. Such an adaptation could serve to maximize bilinguals' access to important social and linguistic cues like audiovisual congruence (Mercure et al. 2019), non‐speech facial information (Fort et al. 2017), and language‐specific articulatory information found on the mouth (Sebastián‐Gallés et al. 2012; Weikum et al. 2007). López‐Pérez et al. (2020) found that receptive language scores at 24 months were associated with visual fixation patterns on Face Pop‐Out at 6 to 7 months. Specifically, they reported a negative link between refixation on areas of Face Pop‐Out slides at 6 to 7 months and receptive language skills at 24 months. Instead, visiting more object areas on the screen was statistically predictive of later receptive language skills. This indicates that increased drive to explore novel non‐social objects, facilitated by a freeing up of attention after initial orientation to the face, might support early language development in the first 2 years of life (López‐Pérez et al. 2020). The current finding adds to this result by suggesting that bilingual infants show significantly stronger propensity to disengage their attention from faces and explore other objects than monolinguals, which could constitute a bilingual‐specific adaptation in visual strategy that supports bilinguals' dual‐language learning goals. However, future research is required to replicate the longitudinal link between visual attention patterns shown here and language development, and to determine if this relationship is quantitatively different in children learning one language compared to those learning two.

The data here also revealed that, regardless of variability in children's early language experiences, dynamic allocation of attention to static faces showed strong developmental shifts within age groups (see Supporting Information S1: Tables S11 and S12 for infants and Supporting Information S1: Tables S16 and S17 for toddlers). In both age groups, younger children looked more to the face overall than older children, and the timing of attentional trajectories were age sensitive. In infants, younger children showed a stronger re‐capture by the face earlier in the trial compared to older children, who showed a later return to the face. In contrast, toddlers exhibited a gradual decline in attention, with later return to the face. Broadly, two interconnected neurocognitive processes are thought to support attention to faces (Morton and Johnson 1991; M. H. Johnson et al. 2015): a subcortical pathway present at birth but active across the lifespan (Burra et al. 2014; Senju and Johnson 2009) that supports reflexive orientation to faces early in life, and a later‐to‐mature cortical network specialized for processing the information faces provide (e.g., about a person's emotional state or identity, M. Johnson 2005, 2011; M. H. Johnson et al. 2015). The age‐modulated trajectories in infant and toddlers' attention to faces found here are by no means direct evidence for either mechanism; however, they are consistent with previous findings that suggest faces automatically capture attention over non‐faces (e.g., Gliga et al. 2009; Elsabbagh et al. 2013; Mercure et al. 2018; Mousley et al. 2023) and that later looking to faces might reflect the social relevance of the face or ongoing information processing (Frank et al. 2011; Gliga and Csibra 2007; Mirman et al. 2008). Another study found that 3‐ to 9‐month‐old infants' looking to faces in animated videos increased with age (Frank et al. 2009). This finding was replicated by Frank et al. (2014), who also showed that infants' amount of face looking was correlated with infants' performance on a visual search task. A longitudinal study by Tomalski et al. (2021) also reported that children show more sequential fixations, consecutively fixating on the same location (e.g., more in‐depth scanning of one area), and a higher overall percentage of re‐fixations (e.g., revisiting of previously fixated areas) when viewing social (but not non‐social) stimuli at 11 months than they did at 5.5 months. The age patterns found here align with Tomalski et al. (2021), suggesting that visual scanning patterns for social information may become more complex and more structured with age.

### 4.2 Dynamic Video and Mouth Looking

All participants demonstrated structured gaze patterns toward the mouth; however, key differences emerged in how and when children engaged with the mouth based on their age and their language backgrounds. 7‐ to 18‐month‐old infants showed an early peak in mouth‐looking followed by a gradual decline that was particularly strong in bilinguals (see Table 2 and Figure 4A). This pattern mirrors bilinguals' stronger disengagement from faces compared to monolinguals in the same age group (see Table 2). Young infants — especially those growing up learning multiple languages — may extract visual information available on the mouth early in viewing, then shift attention elsewhere as the trial progresses. The bilingual disengagement effect for mouths might be interpreted in much the same way as for faces (see discussion of López‐Pérez et al. 2020 above). Between 7 and 18 months, effects of bilingualism on the temporal structuring of social attention, both to faces and to mouths, may be characterized by stronger disengagement in bilinguals than monolinguals after a period of initial orientation. This could permit bilinguals to more rapidly forage other areas of the visual field for relevant information. However, 18–34 months‐olds with bilingual mothers (but not with monolingual mothers) showed a gradual increase in mouth‐looking over stimulus time. A similar pattern was reported in a 5‐year‐old sample by Birulés et al. (2024), who found that Catalan‐Spanish bilinguals looked more to mouths from the start to the end of audiovisual speech production trials than Spanish monolinguals. Future studies might investigate within‐trial shifts in mouth looking in relation to target activation trajectories seen in linguistic processing (see Supporting Information S1: Table S28 and Figure 4; Mirman et al. 2008). Another study by Tsang et al. (2018) found that relative preference for the mouth over the eyes was positively related to expressive language skills of 6‐ to 12‐month‐old monolingual and bilingual children. As children develop, they may become more deliberate in directing attention to the mouth to support their specific language processing demands, rather than relying on an early, reflexive preference.

Despite clear language group differences in mouth‐looking strategies, similarly to face‐looking, within‐group bilingual infant analyses showed no significant relationship between infants' degree of bilingual exposure or parental language mixing and gaze patterns (see Supporting Information S1: Table S24). Mouth looking in the Dynamic Video were also not related to bilingual infants' percentage of exposure to English, which was the language used in the video when speech occurred (see Supporting Information S1: Table S24). It might be that bilingual effects on mouth‐looking are driven by broad developmental adaptation, rather than one that is directly shaped by fine‐grained individual differences in exposure. Indeed, even a small amount of second language exposure (e.g., 5% Spanish/95% English) constitutes many hours of second language exposure for a bilingual child and may be enough for children to recognize the complexity of their language environment (Kuhl et al. 2003). Even a relatively small degree of bilingual language exposure could trigger attentional adaptations regardless of the extent to which their input is balanced between their two languages.

At an average level, toddlers with a bilingual mother looked more to mouths overall than those with a monolingual mother, an effect that was not present in bilingual compared to monolingual infants. Previous literature on group effects in overall mouth‐looking are mixed: for example, Pons et al. (2015) report more mouth‐looking in bilinguals than monolinguals at ages 4 and 12 months, whilst others do not report group differences at 7–10 months (Mercure et al. 2022) nor at 5 months, 9 months, 12 months, 14 months, 2 years, 3 years, or 4–5 years (Morin‐Lessard et al. 2019). It might be that divergent findings are related to methodological precision. In the present study, some analyses revealed that bilinguals' attentional trajectories to faces over stimulus time differed significantly from monolinguals even when average‐level group differences were absent (see Table 2). Where traditional statistical methods do not report average‐level effects, it may be important to consider whether retaining time‐series information allows for a more suitable test of effects of interest. Maximizing analytical granularity is particularly useful where effects may be inconsistent or hard to detect, as is often the case in developmental eye‐tracking research. Where average‐level group differences are reported, time series techniques can provide insight into what underlying dynamics might drive them. For example, in the present study, toddlers with bilingual mothers look more to mouths than those with monolingual mothers overall. Modeling of temporal profiles across stimulus time suggests this average‐level difference is driven by those with bilingual mothers showing a sharper linear increase in mouth‐attention over time than those with monolingual mothers.

### 4.3 Limitations and Future Directions

It is important to highlight that the toddlers in this study were classified according to their mothers' first language, either as English‐speaking monolinguals if their first language was English, or as bilingual users of English and a second non‐English language if their first language was not English. This was the best measure of likely bilingual experience available in the toddler cohort but, because the data was made available from a study that was not designed to test language group differences, it was not possible to characterize toddlers' own degree of exposure to a non‐English language. Participants in the infant group were recruited specifically for studies investigating early experiences of bilingualism, and in‐depth language input profiles were available for these children. Given this, language group effects should be interpreted within age groups (i.e., infants separately from toddlers) and should not be considered direct evidence of developmental effects of bilingualism across the full age range. For example, bilinguals showed more rapid disengagement from faces compared to monolinguals in the infant group, but this result was not present in toddler group. It could be that bilingual effects diminish with age or that the imprecise classification of bilingualism in toddlers obscures a meaningful effect that is truly present. The results of this study cannot be interpreted either way. Future research might seek to clarify this point.

It is also important to highlight that bilingualism exists on a continuum, and debate exists about the validity of categorical group distinction between bilinguals versus monolinguals (Kremin and Byers‐Heinlein 2021). Here, for the sake of continuity given the limited information available about toddlers' early language input, the present study implemented categorical group distinction in both samples. For infants with direct measures of bilingual experience, when differences across language groups emerged, within‐group effects in bilinguals (i.e., degree of bilingualism and language mixing) were explored. Future research which quantifies bilingual experience as a continuous variable may be better suited to identify what drives bilingual‐specific effects. This is particularly important given that sources of within‐group variability have been shown to play a part in bilinguals' early social attention. For example, bilinguals' attention to speakers' mouths is sensitive to the distance, or the degree of difference, between their two native language pairs. In 4‐ to six‐month‐olds and 15‐month‐olds, Birulés et al. (2018) report that close‐language bilinguals showed a stronger preference for the mouth over the eyes than distant‐language bilinguals. It might be that bilinguals learning two languages which are similar to each other (e.g., Spanish and Catalan) rely more on the redundant audiovisual speech cues of a speaker's mouth than do those learning two more distant languages (e.g., Spanish and Russian). The present study was not designed to test effects of language distance and instead took a somewhat opposite approach, testing between‐group differences in monolinguals compared to a large, heterogenous group of children with bilingual experience or with a bilingual mother (see Figure 1). The strength of this approach is that the effects reported here emerge across a wide variety of early bilingual experiences. Post‐hoc analyses revealed no differences based on broadly defined language pair distances (see Supporting Information S1: Tables S41–S45). These findings would be complemented by future investigations designed to test language distance effects on bilinguals' temporal profiles of attention to mouths a priori.

The nature of the Dynamic Video task should also be considered. The stimuli contained more variability than is typical in bilingual eye‐tracking studies, which tend to use stimuli in which number of speakers, orientation and movement of the face, and auditory input are strictly controlled (e.g., Birulés et al. 2018, 2023; Pons and Lewkowicz (2014). Pons et al. 2015, 2019). The benefit of using a relatively natural stimulus is that effects present are perhaps more reflective of a child's visual attention in “real world” viewing of complex and dynamic social scenes, which often contain multiple people and background noise. Of course, future research on visual attention in naturalistic social interaction is required to determine the generalizability of age and language group differences in attention to mouths. Future research, particularly in naturalistic interaction, might also investigate differences in looking to speaking and non‐speaking mouths over time. The faces in the Dynamic Video spoke for 60.25% of the total trial time. The consequence is that infants' and toddlers' looking to mouths in this study cannot be interpreted as looking to mouths which were only ever speaking. In a naturalistic situation, it is not common for an interlocutor to speak continuously throughout an interaction. The stimuli here, which spoke for a portion of stimulus time, may thus increase ecological validity.

When faces were speaking, they were doing so in English, a language with which all children had some degree of previous experience. A viewer's experience with a stimulus language plays a part in their audiovisual speech processing. One study of five‐year‐old children found differences in looking to mouths of native versus non‐native audiovisual speech in Spanish‐Catalan bilinguals and Spanish monolinguals (Birulés et al. 2023). Whilst watching non‐native (English) speech, both monolingual and bilingual children looked more to mouths than eyes but, later into stimulus time, showed no preference to mouths versus eyes. When viewing native speech (Spanish), monolinguals looked equally to eyes and mouths throughout the trial, whilst bilinguals attended more to the mouth than eyes initially before attending equally to the eyes and mouths later into stimulus time (Birulés et al. 2023). Future studies might aim to test the age at which monolingual versus bilinguals' preference to mouths over eyes of faces producing native speech.

Finally, to the authors' knowledge, there was no published evidence about experience‐sensitive differences in the shape of temporal trajectories of attention to faces or mouths at the time of this study's pre‐registration. It was planned a priori that model polynomials would be used to explore the predicted group differences, but no specific predictions were made about which polynomial components (e.g., linear, quadratic, cubic) would differ, or in which direction, between the two groups in the absence of previous literature. Implementation of time‐series analyses to characterize social attention trajectories in large, diverse samples of monolingual and bilingual children across the early years will allow for increasingly specific a priori hypotheses about how experience may modulate the shape of visual attention trajectories.

## 5 Conclusion

Whether bilinguals show experience‐related adaptations in social attention is a popular scientific question, but studies often contend with the same simple but challenging methodological issue: testing the hypothesis of visual strategy differences requires measuring visual strategies. Most research reports aggregate‐level group differences in data averaged across a specified duration (e.g., Ayneto and Sebastián‐Gallés 2017; Birulés et al. 2018; Pons et al. 2015, 2019; Mercure et al. 2018; Mousley et al. 2023). These studies analyze measures such as total looking time, total number of fixations, or constructs like proportion of total looking time and average dwell time that rely on average measures. Such metrics provide important indications about where bilingual effects might exist, but they cannot explain what visual strategies drive effects of interest. Using growth curve analysis, the present study provides a novel exploration of fine‐grained shifts in attention over stimulus time, providing more room for strategy‐related interpretation than is typical. The results suggest that temporal dynamics of social attention are sensitive both to age and to children's early language environments. Between 7 and 18 months), temporal patterns of visual attention are characterized by a propensity for visual scavenging of social stimuli, which is stronger in bilinguals than monolinguals for both static faces and dynamic mouths. Age‐dependent shifts are evident, with younger infants re‐fixating on static faces earlier in time than older infants, who show later return. Within the group of toddlers, attention to faces is more stable over time in older than younger children, as older toddlers showed a more gradual initial decline, a weaker late return, and less net decline overall than younger toddlers. Mouth‐looking shows fewer age‐driven shifts than face‐looking and is instead more affected by children's early language experience. Younger bilingual infants show more rapid disengagement from mouths than monolingual infants, a pattern that appears in reverse in toddlers aged 18–34 months, where those with a bilingual mother show stronger attentional allocation to mouths over time than those with a monolingual mother. The results are consistent with the hypothesis that bilinguals might show differences in social attention compared to monolinguals, potentially explained by a drive to maximize their access to the social and linguistic information they need to support their dual‐language learning goals. The results also align with previous findings suggesting there are developmental shifts in the re‐organization of young children's visual attention for viewing of static and dynamic social stimuli.

## Author Contributions

**Victoria St. Clair:** conceptualization, data curation, formal analysis, funding acquisition, project administration, visualization, investigation, writing – original draft, methodology. **Teresa Del Bianco:** conceptualization, data curation, formal analysis, methodology, project administration, writing – review and editing. **Emily J. H. Jones:** conceptualization, project administration, supervision, writing – review and editing, methodology. **Mairéad MacSweeney:** supervision, project administration, writing – review and editing. **Roberto Filippi:** funding acquisition, project administration, writing – review and editing. **Peter Bright:** funding acquisition, project administration, writing – review and editing. **Atsushi Senju:** project administration, writing – review and editing. **Evelyne Mercure:** conceptualization, methodology, project administration, supervision, writing – review and editing.

## Funding

Victoria St. Clair was supported by a University College London Research Excellence Scholarship and by the Leverhulme Trust (RPG‐2021‐280). Mairéad MacSweeney was supported by Wellcome Trust Senior Research Fellowships (100229/Z/12/Z and 220291/Z/20/Z). Evelyne Mercure was supported by an Economic and Social Research Council (ESRC) Future Research Leader fellowship (ES/K001329/1). This work was also supported by a British Academy/Leverhulme grant (SG1621171) awarded to Roberto Filippi and Peter Bright. The Developing Human Connectome Project was funded by the European Research Council under the European Union Seventh Framework Program (FP/20072013)/ERC Grant Agreement no. 319456.

## Ethics Statement

Data from infants were collected as part of two studies approved by both UCL and Birkbeck ethics committees. Data from toddlers were collected by the *Developing Human Connectome Project* which was approved by the United Kingdom National Research Ethics Authority.

## Conflicts of Interest

The authors declare no conflicts of interest.

## Supporting information


Supporting Information S1

## Data Availability

Data from infants will be made available upon reasonable request for participants whose parents provided consent for their child's information to be shared externally. Data from toddlers are publicly available at: https://biomedia.github.io/dHCP‐release‐notes/.

## References

[infa70077-bib-0001] Ayneto, A. , and N. Sebastián‐Gallés . 2017. “The Influence of Bilingualism on the Preference for the Mouth Region of Dynamic Faces.” Developmental Science 20, no. 1: e12446. 10.1111/desc.12446. 27196790

[infa70077-bib-0002] Birulés, J. , L. Bosch , D. J. Lewkowicz , and F. Pons . 2024. “Time Course of Attention to a Talker’s Mouth in Monolingual and Close‐Language Bilingual Children.” Developmental Psychology 60, no. 1: 135–143. 10.1037/dev0001659. 37917490

[infa70077-bib-0003] Birulés, J. , L. Bosch , R. Brieke , F. Pons , and D. J. Lewkowicz . 2018. “Inside Bilingualism: Language Background Modulates Selective Attention to a Talker’s Mouth.” Developmental Science 22, no. 3: e12755. 10.1111/desc.12755. 30251757

[infa70077-bib-0067] Birulés, J. , L. Goupil , J. Josse , and M. Fort . 2023. “The Role of Talking Faces in Infant Language Learning: Mind the Gap Between Screen‐Based Settings and Real‐Life Communicative Interactions.” Brain Sciences 13, no. 8: 1167. 10.3390/brainsci13081167. 37626523 PMC10452843

[infa70077-bib-0004] Bosch, L. , and N. Sebastián‐Gallés . 1997. “Native‐Language Recognition Abilities in 4‐Month‐Old Infants From Monolingual and Bilingual Environments.” Cognition 65, no. 1: 33–69. 10.1016/s0010-0277(97)00040-1. 9455170

[infa70077-bib-0005] Brainard, D. H. 1997. “The Psychophysics Toolbox.” Spatial Vision 10, no. 4: 433–436. 10.1163/156856897x00357. 9176952

[infa70077-bib-0006] Burra, N. , D. Kerzel , B. de Gelder , and A. J. Pegna . 2014. “Lack of Automatic Attentional Orienting by Gaze Cues Following a Bilateral Loss of Visual Cortex.” Neuropsychologia 58: 75–80. 10.1016/j.neuropsychologia.2014.04.003. 24732381

[infa70077-bib-0007] Byers‐Heinlein, K. 2013. “Parental Language Mixing: Its Measurement and the Relation of Mixed Input to Young Bilingual Children’s Vocabulary Size.” Bilingualism: Language and Cognition 16, no. 1: 32–48. 10.1017/S1366728912000120.

[infa70077-bib-0008] Byers‐Heinlein, K. 2015. “Methods for Studying Infant Bilingualism.” In The Cambridge Handbook of Bilingual Processing, edited by J. W. Schweiter , 133–154. 10.1017/CBO9781107447257.005.

[infa70077-bib-0009] Byers‐Heinlein, K. , T. C. Burns , and J. F. Werker . 2010. “The Roots of Bilingualism in Newborns.” Psychological Science 21, no. 3: 343–348. 10.1177/0956797609360758. 20424066

[infa70077-bib-0010] Byers‐Heinlein, K. , A. Jardak , E. Fourakis , and C. Lew‐Williams . 2020. “Effects of Language Mixing on Bilingual Children’s Word Learning.” PsyArXiv. 10.31234/osf.io/298cz. PMC8992731 35399292

[infa70077-bib-0011] Carbajal, M. J. , and S. Peperkamp . 2020. “Dual Language Input and the Impact of Language Separation on Early Lexical Development.” Infancy: The Official Journal of the International Society on Infant Studies 25, no. 1: 22–45. 10.1111/infa.12315. 32749052

[infa70077-bib-0012] Chandrasekaran, C. , A. Trubanova , S. Stillittano , A. Caplier , and A. A. Ghazanfar . 2009. “The Natural Statistics of Audiovisual Speech.” PLoS Computational Biology 5, no. 7: e1000436. 10.1371/journal.pcbi.1000436. 19609344 PMC2700967

[infa70077-bib-0013] DeAnda, S. , L. Bosch , D. Poulin‐Dubois , P. Zesiger , and M. Friend . 2016. “The Language Exposure Assessment Tool: Quantifying Language Exposure in Infants and Children.” Journal of Speech, Language, and Hearing Research 59, no. 6: 1346–1356. 10.1044/2016_JSLHR-L-15-0234. PMC5399762 27784032

[infa70077-bib-0014] Del Bianco, T. , L. Mason , T. Charman , et al. 2021. “Temporal Profiles of Social Attention Are Different Across Development in Autistic and Neurotypical People.” Biological Psychiatry: Cognitive Neuroscience and Neuroimaging 6, no. 8: 813–824. 10.1016/j.bpsc.2020.09.004. 33191160

[infa70077-bib-0015] Del Bianco, T. , L. Mason , M. Lai , et al. 2022. “Unique Dynamic Profiles of Social Attention in Autistic Females.” Journal of Child Psychology and Psychiatry 63, no. 12: 1602–1614. 10.1111/jcpp.13630. 35634865 PMC9796530

[infa70077-bib-0016] D’Souza, D. , D. Brady , J. Haensel , and H. D’Souza . 2020. “Is Mere Exposure Enough? The Effects of Bilingual Environments on Infant Development.” Royal Society Open Science 7, no. 2: 180191. 10.1098/rsos.180191. 32257297 PMC7062077

[infa70077-bib-0017] Edwards, D. A. , D. Rueckert , S. M. Smith , et al. 2022. “The Development Human Connectome Project Neonatal Data Release.” Frontiers in Neuroscience 16: 886772. 10.3389/fnins.2022.886772. 35677357 PMC9169090

[infa70077-bib-0018] ELAN 2019. Version 5.8. [Computer software], Max Planck Institute for Psycholinguistics, The Language Archive. https://archive.mpi.nl/tla/elan.

[infa70077-bib-0019] Elsabbagh, M. , T. Gliga , A. Pickles , K. Hudry , T. Charman , and M. H. Johnson . 2013. “The Development of Face Orienting Mechanisms in Infants at‐risk for Autism.” Behavioural Brain Research 251: 147–154. 10.1016/j.bbr.2012.07.030. 22846849 PMC3730054

[infa70077-bib-0020] Fort, M. , A. Ayneto‐Gimeno , A. Escrichs , and N. Sebastián‐Gallés . 2017. “Impact of Bilingualism on Infants’ Ability to Learn From Talking and Nontalking Faces.” Supplement, Language Learning 68, no. S1: S31–S57. 10.1111/lang.12273.

[infa70077-bib-0021] Frank, M. C. , D. Amso , and S. P. Johnson . 2014. “Visual Search and Attention to Faces in Early Infancy.” Journal of Experimental Child Psychology 31, no. 9: 1713–1723. 10.1109/TMI.2012.2196707. PMC3844087 24211654

[infa70077-bib-0022] Frank, M. C. , E. Vul , and S. P. Johnson . 2009. “Development of Infants’ Attention to Faces During the First Year.” Cognition 110, no. 2: 160–170. 10.1016/j.cognition.2008.11.010. 19114280 PMC2663531

[infa70077-bib-0023] Frank, M. C. , E. Vul , and R. Saxe . 2011. “Measuring the Development of Social Attention Using Free‐Viewing.” Infancy: The Official Journal of the International Society on Infant Studies 17, no. 4: 355–375. 10.1111/j.1532-7078.2011.00086.x. 32693486

[infa70077-bib-0024] Gliga, T. , and G. Csibra . 2007. “Seeing the Face Through the Eyes: A Developmental Perspective on Face Expertise.” Progress in Brain Research 164: 323–339. 10.1016/S0079-6123(07)64018-7. 17920440

[infa70077-bib-0025] Gliga, T. , M. Elsabbagh , A. Andravizou , and M. Johnson . 2009. “Faces Attract Infants’ Attention in Complex Displays.” Infancy: The Official Journal of the International Society on Infant Studies 14, no. 5: 550–562. 10.1080/15250000903144199. 32693531

[infa70077-bib-0026] Gluckman, M. , and S. P. Johnson . 2013. “Attentional Capture by Social Stimuli in Young Infants.” Frontiers in Psychology 4: 527. 10.3389/fpsyg.2013.00527. 23966966 PMC3744870

[infa70077-bib-0027] Grosjean, F. 2010. Bilingual: Life and Reality. Harvard University Press. 10.4159/9780674056459.

[infa70077-bib-0028] Guarnera, M. , P. Magnano , M. Pellerone , M. I. Cascio , V. Squatrito , and S. L. Buccheri . 2018. “Facial Expressions and the Ability to Recognize Emotions From the Eyes or Mouth: A Comparison Among Old Adults, Young Adults, and Children.” Journal of Genetic Psychology 179, no. 5: 297–310. 10.1080/00221325.2018.1509200. 30346916

[infa70077-bib-0029] Hoff, E. , C. Core , S. Place , R. Rumiche , M. Señor , and M. Parra . 2012. “Dual Language Exposure and Early Bilingual Development.” Journal of Child Language 39, no. 1: 1–27. 10.1017/S0305000910000759. 21418730 PMC4323282

[infa70077-bib-0030] Johnson, M. 2005. “Subcortical Face Processing.” Nature Reviews Neuroscience 6, no. 10: 766–774. 10.1038/nrn1766. 16276354

[infa70077-bib-0031] Johnson, M. 2011. “Face Perception: A Developmental Perspective.” In The Oxford Handbook of Face Perception, edited by A. Calder , G. Rhodes , M. Johnson , and J. Haxby , 3–14. Oxford University Press.

[infa70077-bib-0032] Johnson, M. H. , A. Senju , and P. Tomalski . 2015. “The Two‐Process Theory of Face Processing: Modifications Based on Two Decades of Data From Infants and Adults.” Neuroscience & Biobehavioral Reviews 50: 169–179. 10.1016/j.neubiorev.2014.10.009. 25454353

[infa70077-bib-0033] Kalashnikova, M. , J. Pejovic , and M. Carreiras . 2020. “The Effects of Bilingualism on Attentional Processes in the First Year of Life.” Developmental Science 24, no. 2: e13011. 10.1111/desc.13011. 32603543

[infa70077-bib-0034] Kleiner, M. , D. Brainard , D. Pelli , A. Ingling , R. Murray , and C. Broussard . 2007. “What’s New in Psychtoolbox‐3.” Perception 36: 1–16. https://pure.mpg.de/pubman/faces/ViewItemOverviewPage.jsp?itemId=item_1790332.

[infa70077-bib-0035] Knappmeyer, B. , I. M. Thornton , and H. H. Bülthoff . 2003. “The Use of Facial Motion and Facial Form During the Processing of Identity.” Vision Research 43, no. 18: 1921–1936. 10.1016/S0042-6989(03)00236-0. 12831755

[infa70077-bib-0036] Kremin, L. V. , and K. Byers‐Heinlein . 2021. “Why Not Both? Rethinking Categorical and Continuous Approaches to Bilingualism.” International Journal of Bilingualism 25, no. 6: 1560–1575. 10.1177/13670069211031986. PMC8637352 34867070

[infa70077-bib-0037] Król, M. E. 2018. “Auditory Noise Increases the Allocation of Attention to the Mouth, and the Eyes Pay the Price: An Eye‐Tracking Study.” PLoS One 13, no. 3: e0194491. 10.1371/journal.pone.0194491. 29558514 PMC5860771

[infa70077-bib-0038] Kuhl, P. K. , F.‐M. Tsao , and H.‐M. Liu . 2003. “Foreign‐Language Experience in Infancy: Effects of Short‐Term Exposure and Social Interaction on Phonetic Learning.” Proceedings of the National Academy of Sciences of the United States of America 100, no. 15: 9096–9101. 10.1073/pnas.1532872100. 12861072 PMC166444

[infa70077-bib-0039] Lai, M. , M. V. Lombardo , J. Suckling , et al. 2013. “Biological Sex Affects the Neurobiology of Autism.” Brain 136, no. 9: 2799–2815. 10.1093/brain/awt216. 23935125 PMC3754459

[infa70077-bib-0040] Lewkowicz, D. J. , and A. M. Hansen‐Tift . 2012. “Infants Deploy Selective Attention to the Mouth of a Talking Face When Learning Speech.” Proceedings of the National Academy of Sciences of the United States of America 109, no. 5: 1431–1436. 10.1073/pnas.1114783109. 22307596 PMC3277111

[infa70077-bib-0041] Lewkowicz, D. J. , and K. S. Kraebel . 2004. “The Value of Multisensory Redundancy in the Development of Intersensory Perception.” In The Handbook of Multisensory Processes, edited by G. A. Calvert , C. Spence , and B. E. Stein , 655–678. MIT Press.

[infa70077-bib-0042] López‐Pérez, D. , P. Tomalski , A. Radkowska , H. Ballieux , and D. G. Moore . 2020. “Efficiency of Scanning and Attention to Faces in Infancy Independently Predict Language Development in a Multiethnic and Bilingual Sample of 2‐Year‐Olds.” First Language 41, no. 2: 218–239. 10.1177/0142723720966815.

[infa70077-bib-0043] Mercure, E. , P. Bright , I. Quiroz , and R. Filippi . 2022. “Effect of Infant Bilingualism on Audiovisual Integration in a Mcgurk Task.” Journal of Experimental Child Psychology 217: 105351. 10.1016/j.jecp.2021.105351. 35093667

[infa70077-bib-0044] Mercure, E. , E. Kushnerenko , L. Goldberg , et al. 2019. “Language Experience Influences Audiovisual Speech Integration in Unimodal and Bimodal Bilingual Infants.” Developmental Science 22, no. 1: e12701. 10.1111/desc.12701. 30014580 PMC6393757

[infa70077-bib-0045] Mercure, E. , I. Quiroz , L. Goldberg , et al. 2018. “Impact of Language Experience on Attention to Faces in Infancy: Evidence From Unimodal and Bimodal Bilingual Infants.” Frontiers in Psychology 9: 1943. 10.3389/fpsyg.2018.01943. 30459671 PMC6232685

[infa70077-bib-0046] Mirman, D. , J. A. Dixon , and J. S. Magnuson . 2008. “Statistical and Computational Models of the Visual World Paradigm: Growth Curves and Individual Difference.” Journal of Memory and Language 59, no. 4: 475–494. 10.1016/j.jml.2007.11.006. 19060958 PMC2593828

[infa70077-bib-0047] Morin‐Lessard, E. , D. Poulin‐Dubois , N. Segalowitz , and K. Byers‐Heinlein . 2019. “Selective Attention to the Mouth of Talking Faces in Monolinguals and Bilinguals Aged 5 Months to 5 Years.” Developmental Psychology 55, no. 8: 1640–1655. 10.1037/dev0000750. 31169400

[infa70077-bib-0048] Morton, J. , and M. Johnson . 1991. “CONSEPC and CONLERN: A Two‐Process Theory of Infant Face Recognition.” Psychological Review 98, no. 2: 164–181. 10.1037/0033-295x.98.2.164. 2047512

[infa70077-bib-0049] Mousley, V. L. , M. MacSweeney , and E. Mercure . 2023. “Bilingual Toddlers Show Increased Attention Capture by Static Faces Compared to Monolinguals.” Bilingualism: Language and Cognition 26, no. 4: 835–844. 10.1017/s136672892200092x. 37636491 PMC7614981

[infa70077-bib-0050] O’Donnell, C. , and V. Bruce . 2001. “Familiarisation With Faces Selectively Enhances Sensitivity to Changes Made to the Eyes.” Perception 30, no. 6: 755–764. 10.1068/p3027. 11464563

[infa70077-bib-0051] Pons, F. , L. Bosch , and D. J. Lewkowicz . 2015. “Bilingualism Modulates Infants’ Selective Attention to the Mouth of a Talking Face.” Psychological Science 26, no. 4: 490–498. 10.1177/0956797614568320. 25767208 PMC4398611

[infa70077-bib-0052] Pons, F. , L. Bosch , and D. J. Lewkowicz . 2019. “Twelve‐Month‐Old Infants’ Attention to the Eyes of a Talking Face Is Associated With Communication and Social Skills.” Infant Behavior and Development 54: 80–84. 10.1016/j.infbeh.2018.12.003. 30634137

[infa70077-bib-0053] Pons, F. , and D. J. Lewkowicz . 2014. “Infant Perception of Audio‐Visual Speech Synchrony in Familiar and Unfamiliar Fluent Speech.” Acta Psychologica 149: 142–147. 10.1016/j.actpsy.2013.12.013. 24576508

[infa70077-bib-0054] Potter, C. E. , E. Fourakis , E. Morin‐Lessard , K. Byers‐Heinlein , and C. Lew‐Williams . 2018. “Bilingual Toddlers’ Comprehension of Mixed Sentences Is Asymmetrical Across Their Two Languages.” Developmental Science 22, no. 4: e12794. 10.1111/desc.12794. PMC6570532 30582256

[infa70077-bib-0055] Sebastián‐Gallés, N. , B. Albareda‐Castellot , W. M. Weikum , and J. F. Werker . 2012. “A Bilingual Advantage in Visual Language Discrimination in Infancy.” Psychological Science 23, no. 9: 994–999. 10.1177/0956797612436817. 22810164

[infa70077-bib-0056] Senju, A. , and M. H. Johnson . 2009. “The Eye Contact Effect: Mechanisms and Development.” Trends in Cognitive Sciences 13, no. 3: 127–134. 10.1016/j.tics.2008.11.009. 19217822

[infa70077-bib-0057] Singh, L. 2018. “Bilingual Infants Demonstrate Advantages in Learning Words in a Third Language.” Child Development 89, no. 4: e397–e413. 10.1111/cdev.12852. 28556913

[infa70077-bib-0058] Singh, L. , and A. R. Y. Tan . 2020. “Beyond Perceptual Narrowing: Monolingual and Bilingual Infants Discriminate Hindi Contrasts When Learning Words in the Second Year of Life.” Developmental Psychology 57, no. 1: 19–32. 10.1037/dev0001137. 33271031

[infa70077-bib-0059] Teinonen, T. , R. N. Aslin , P. Alku , and G. Csibra . 2008. “Visual Speech Contributes to Phonetic Learning in 6‐Month‐Old Infants.” Cognition 108, no. 3: 850–855. 10.1016/j.cognition.2008.05.009. 18590910

[infa70077-bib-0060] Tomalski, P. , D. López Pérez , A. Radkowska , and A. Malinowska‐Korczak . 2021. “Selective Changes in Complexity of Visual Scanning for Social Stimuli in Infancy.” Frontiers in Psychology – Developmental Psychology 12: 705600. 10.3389/fpsyg.2021.705600. PMC8593402 34795610

[infa70077-bib-0061] Tsang, T. , N. Atagi , and S. P. Johnson . 2018. “Selective Attention to the Mouth Is Associated With Expressive Language Skills in Monolingual and Bilingual Infants.” Journal of Experimental Child Psychology 169: 93–109. 10.1016/j.jecp.2018.01.002. 29406126 PMC5933852

[infa70077-bib-0062] Tsui, A. S. M. T. , L. C. Erickson , A. Mallikarjunn , E. D. Thiessen , and C. T. Fennell . 2020. “Dual Language Statistical Word Segmentation in Infancy: Simulating a Language‐Mixing Bilingual Environment.” Developmental Science 24, no. 3: e13050. 10.1111/desc.13050. 33063938

[infa70077-bib-0063] Võ, M. L.‐H. , T. J. Smith , P. K. Mital , and J. M. Henderson . 2012. “Do the Eyes Really Have It? Dynamic Allocation of Attention When Viewing Moving Faces.” Journal of Vision 12, no. 13: 1–14. 10.1167/12.13.3. 23211270

[infa70077-bib-0064] Weikum, W. M. , A. Vouloumanos , J. Navarra , S. Soto‐Faraco , N. Sebastián‐Gallés , and J. F. Werker . 2007. “Visual Language Discrimination in Infancy.” Science 316, no. 5828: 1159. 10.1126/science.1137686. 17525331

[infa70077-bib-0065] Werker, J. F. 2012. “Perceptual Foundations of Bilingual Acquisition in Infancy.” Annals of the New York Academy of Sciences 1251, no. 1: 50–61. 10.1111/j.1749-6632.2012.06484.x. 22694186

[infa70077-bib-0066] Werker, J. F. 2018. “Perceptual Beginnings to Language Acquisition.” Applied PsychoLinguistics 39, no. 4: 703–728. 10.1017/S0142716418000152.

