# Supplementary material for: Experience‐Sensitive Effects on Temporal Profiles of Social Attention in Early Childhood
Source: Infancy. 2026 Mar 21;31(2):e70077. doi: 10.1111/infa.70077 (PMC13005695; doi:10.1111/infa.70077)
Supplement: Supplementary file 1 — Supporting Information S1 [file INFA-31-0-s001.docx]

**Method**

**Power Calculations**

**Dynamic Video.** A power calculation for the hypothesised effect of bilingualism on mouth looking was conducted using statistics reported by Pons et al. (2015). In a mixed, repeated measures ANOVA, they entered proportion of total looking time to mouths as the outcome variable with area of interest (AOI, eyes vs mouth) as a within-subjects factor and two between-subjects factors: language background (monolingual vs bilingual) and age (four, eight, or 12 months). There was a trend of an effect of a three-way interaction between AOI x Language Background x Age with a reported partial eta squared of .041. This effect size estimate was used to calculate an a priori power analysis in G*Power (version 3.1, Faul et al., 2007), with the following parameters: a statistical test of ‘ANOVA: Repeated measures, within-between interaction’, effect size *f* = .207 (derived from the partial eta squared estimate of .041), alpha error probability = 0.05, for two groups contributing four measurements with correlation among repeated measures = 0.5, and a nonsphericity correction = 1. The result suggests that a total sample size of *N* = 52 would be required to detect an effect of this size using this statistical approach. We therefore expect that we will have sufficient power in both age groups (infants: *n* = 131; toddlers: *n* = 745) to detect an effect size of interest.

**Face Pop-Out.** The effect of bilinguals’ increased attention to faces compared to monolinguals reported by Mousley et al. (2023) was used for a power calculation for the effect expected of bilinguals’ increased attention to static faces compared to monolinguals. Following a significant interaction in a 2 (AOI: face vs non-face) x 2 (monolingual vs bilingual) mixed effects ANOVA, Mousley et al. (2023) used a post-hoc *t*-test to compare the two groups’ overall speed of looking to the face area of the face pop-out slides. Results revealed a significant difference whereby bilinguals’ first look to faces on the slide was significant faster than monolinguals’ (*t*(55.91) = 2.02, *p* = .049, *d* = -0.52). The reported effect size was used to calculate an a priori power analysis in G*Power (version 3.1, Faul et al., 2007), with the following parameters: a statistical test of ‘Means: Difference between two independent means (two groups)’, effect size *d* = 0.52, for a two-tailed test with an alpha error probability rate of 0.05. The result suggests that a total sample size of *N* = 99 would be required to detect an effect of this size using this statistical approach. We expect that we will have sufficient power to detect this effect in the toddler age group (*n* = 745).

**Figure S1.** Visualisation of age distribution by sample.

**
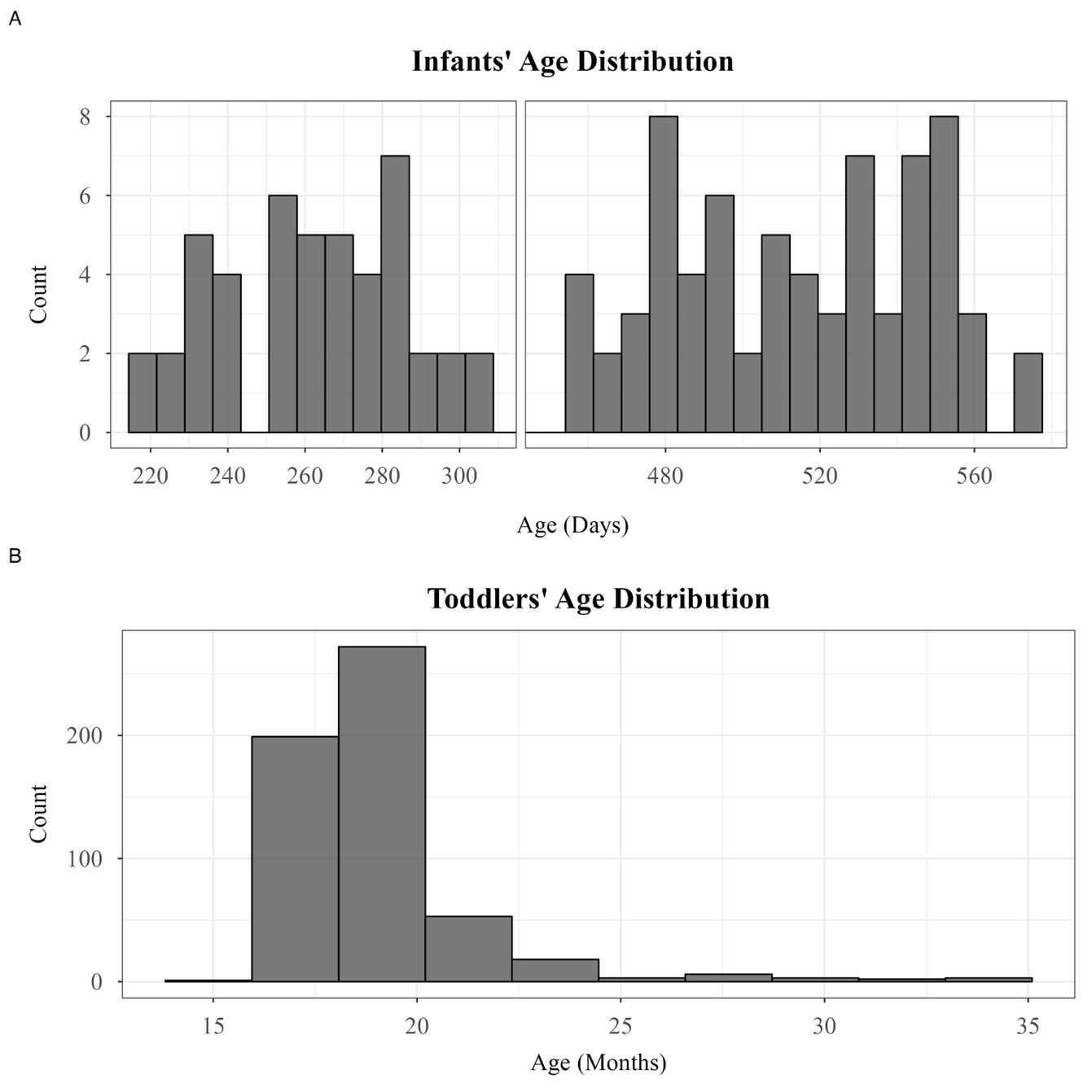
**

| **A** | **Highest Level of Education** | **Mother** | | **Father** | | **Parent Ethnicity** | **Mother** | | | **Father** | |
| --- | --- | --- | --- | --- | --- | --- | --- | --- | --- | --- | --- |
|  |  | **M** | **B** | **M** | **B** |  | **M** | **B** | | **M** | **B** |
|  | GCSE or O-Level | *n* = 0 | *n* = 0 | *n* = 0 | *n* = 3 | White | *n* = 35 | *n* = 21 | | *n* = 29 | *n* = 22 |
|  | A Level or Diploma | *n* = 2 | *n* = 4 | *n* = 6 | *n* = 3 | Asian or Asian British | *n* = 1 | *n* = 3 | | *n* = 1 | *n* = 4 |
|  | Degree or HND | *n* = 27 | *n* = 17 | *n* = 27 | *n* = 21 | Black, African, Caribbean, or Black British | *n* = 3 | *n* = 2 | | *n* = 4 | *n* = 0 |
|  | Postgraduate degree (e.g., MSc) | *n* = 29 | *n* = 30 | *n* = 29 | *n* = 22 | Multiple or mixed ethnic groups | *n* = 1 | *n* = 1 | | *n* = 1 | *n* = 1 |
|  | Doctorate | *n* = 10 | *n* = 4 | *n* = 4 | *n* = 3 | Other ethnic group | *n* = 1 | *n* = 2 | | *n* = 4 | *n* = 1 |
|  | NA | *n* = 4 | *n* = 4 | *n* = 6 | *n* = 7 | NA | *n* = 31 | *n* = 30 | | *n* = 33 | *n* = 31 |
| **B** | **Pre-Tax Household Income** | | | | | | **Language Group** | | | | |
|  |  |  |  |  |  |  | **M** | | **B** | | |
|  | £ 17,001 - £ 20,000 | | | | | | *n* = 0 | | *n* = 1 | | |
|  | < £ 20,000 | | | | | | *n* = 0 | | *n* = 2 | | |
|  | £ 20,000 - £ 29,999 | | | | | | *n* = 3 | | *n* = 5 | | |
|  | £ 25,001 - £ 30,000 | | | | | | *n* = 1 | | *n* = 0 | | |
|  | £ 30,000 - £ 39,999 | | | | | | *n* = 1 | | *n* = 3 | | |
|  | £ 30,001 - £ 40,000 | | | | | | *n* = 3 | | *n* = 1 | | |
|  | £ 40,000 - £ 59,999 | | | | | | *n* = 5 | | *n* = 5 | | |
|  | £ 40,001 - £ 50,000 | | | | | | *n* = 1 | | *n* = 0 | | |
|  | £ 50,000 - £ 90,000 | | | | | | *n* = 1 | | *n* = 0 | | |
|  | £ 50,001 - £ 70,000 | | | | | | *n* = 1 | | *n* = 2 | | |
|  | £ 60,000 - £ 79,999 | | | | | | *n* = 2 | | *n* = 2 | | |
|  | £ 70,001 - £ 90,000 | | | | | | *n* = 6 | | *n* = 7 | | |
|  | £ 80,000 - £ 99,999 | | | | | | *n* = 7 | | *n* = 9 | | |
|  | £ 90,001 - £ 120,000 | | | | | | *n* = 5 | | *n* = 3 | | |
|  | £ 100,000 - £ 149,999 | | | | | | *n* = 9 | | *n* = 6 | | |
|  | > £ 120,000 | | | | | | *n* = 9 | | *n* = 5 | | |
|  | > £ 149,999 | | | | | | *n* = 9 | | *n* = 2 | | |
|  | Don’t know | | | | | | *n* = 2 | | *n* = 2 | | |
|  | Did not answer | | | | | | *n* = 7 | | *n* = 4 | | |

**Table S1.** Participant demographic information (infants only).

**Note.** M = monolingual, B = bilingual. Pre-tax household income and parents’ highest level of education available for all infants (*n* = 131). A chi-square test revealed no differences between the groups (household income: *χ²* = 19.58, *p* = .420; mother education level: *χ²* = 4.28, *p* = .370; father education level: *χ²* = 4.69, *p* = .455). Ethnicity only collected for *n* = 85 infants. Household income collected on two different scales for each study which contributed data, resulting in overlapping income categories. For toddler cohort, ethnicity and parent education was collected but not accessible via public repository at the time of this manuscript’s revision. The NIH website cites a lapse in government funding resulting in a lack of technical support and access request processing.

**Results**

**Figure S2.** Visual representation of temporal trends from Del Bianco et al. 2021.

**
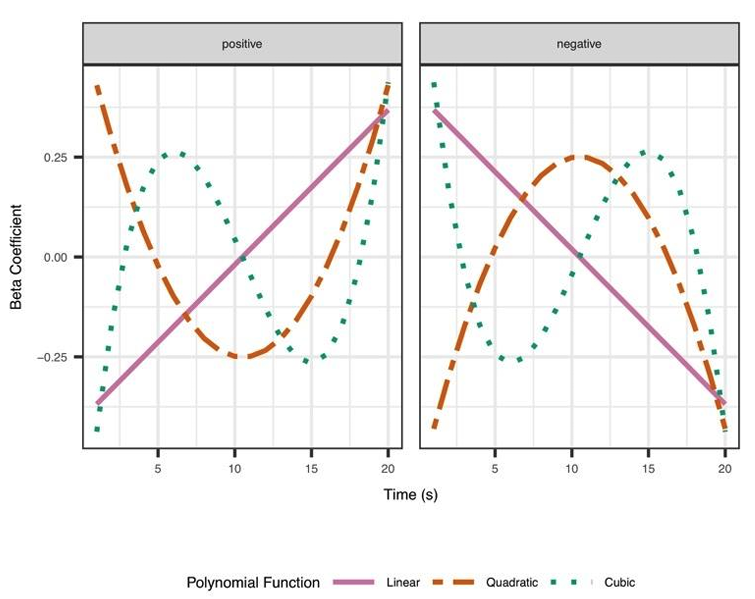
**

**Note.** Figure from Del Bianco et al. (2021) showing: “the visual representation of temporal trends as indexed by the orthogonal polynomial of time: linear slope (pink line), quadratic component (orange dashed), and cubic component (green dotted). A significant beta coefficient (positive or negative) for any of these 3 polynomials is representative of a predominant trend as represented in the figure.”

**Quality Checks**

**Table S2.** Infants, Quality Check – Face Pop-Out Missing Data: Model Selection Output

| **Model** | **N Parameters** | **AIC** | **BIC** | **Deviance** | **Statistic** | **DF** | ***P*-value** |
| --- | --- | --- | --- | --- | --- | --- | --- |
| Base | 3 | 41802 | 41821 | 41796 | - | - | - |
| + Age | 4 | 41802 | 41828 | 41794 | 1.50 | 1 | .220 |
| + Gender | 5 | 41804 | 41836 | 41794 | 0.26 | 1 | .614 |
| + Language Group | 6 | 41805 | 41844 | 41793 | 1.05 | 1 | .306 |

**Note.** Missing data available only from the *n* = 85 of 131 infants. Base model: *missing data ~ (1 | id)*. ‘+’ marks indicate addition of specified variable as a fixed effect. A *p*-value < 0.05 indicates significant model improvement (i.e., better explanatory power) compared to the base model.

**Table S3.** Toddlers, Quality Check – Face Pop-Out Missing Data: Model Selection Output

| **Model** | **N Parameters** | **AIC** | **BIC** | **Deviance** | **Statistic** | **DF** | ***P*-value** |
| --- | --- | --- | --- | --- | --- | --- | --- |
| Base | 3 | 340159 | 340185 | 340153 | - | - | - |
| + Age | 4 | 340161 | 340195 | 340153 | 0.28 | 1 | .598 |
| + Gender | 5 | 340159 | 340202 | 340149 | 3.71 | 1 | .054 |
| *Categorical group predictor* | | | | | | | |
| + Language Group | 6 | 340159 | 340211 | 340147 | 2.16 | 1 | .142 |
| *Numeric group predictor* | | | | | | | |
| + Language Group | 6 | 340160 | 340211 | 340148 | 1.45 | 1 | .228 |

**Note.** Base model: *missing data ~ ( 1 | id)*. ‘+’ marks indicate addition of specified variable as a fixed effect. A *p*-value < 0.05 indicates significant model improvement (i.e., better explanatory power) compared to the base model. Categorical group predictor calculated as monolingual if toddlers’ mothers’ first language was English, bilingual if it was not English. Numeric group predictor calculated as 0 if both parents’ first languages were English, 1 if one parent’s was English, and 2 if neither were English.

**Table S4.** Infants, Quality Check – Dynamic Video Missing Data: Model Selection Output

| **Model** | **N Parameters** | **AIC** | **BIC** | **Deviance** | **Statistic** | **DF** | ***P*-value** |
| --- | --- | --- | --- | --- | --- | --- | --- |
| Base | 3 | -44150 | -44128 | -44156 | - | - | - |
| + Age | 4 | -44148 | -44119 | -44156 | 0.11 | 1 | .739 |
| + Gender | 5 | -44148 | -44112 | -44158 | 1.64 | 1 | .200 |
| + Language Group | 6 | -44148 | -44106 | -44160 | 2.73 | 1 | .099 |

**Note.** Base model: *missing data ~ (1 | id)*. ‘+’ marks indicate addition of specified variable as a fixed effect. A *p*-value < 0.05 indicates significant model improvement (i.e., better explanatory power) compared to the base model.

**Table S5.** Toddlers, Quality Check – Dynamic Video Missing Data: Model Selection Output

| **Model** | **N Parameters** | **AIC** | **BIC** | **Deviance** | **Statistic** | **DF** | ***P*-value** |
| --- | --- | --- | --- | --- | --- | --- | --- |
| Base | 3 | -21911 | -21886 | -21917 | - | - | - |
| + Age | 4 | -21909 | -21876 | -21917 | 0.09 | 1 | .760 |
| + Gender | 5 | -21907 | -21866 | -21917 | 0.09 | 1 | .768 |
| *Categorical group predictor* | | | | | | | |
| + Language Group | 6 | -21905 | -21855 | -21917 | 0.00 | 1 | .987 |
| *Numeric group predictor* | | | | | | | |
| + Language Group | 6 | -21906 | -21856 | -21918 | 0.33 | 1 | .565 |

**Note.** Base model: *missing data ~ (1 | id)*. ‘+’ marks indicate addition of specified variable as a fixed effect. A *p*-value < 0.05 indicates significant model improvement (i.e., better explanatory power) compared to the base model. Categorical group predictor calculated as monolingual if toddlers’ mothers’ first language was English, bilingual if it was not English. Numeric group predictor calculated as 0 if both parents’ first languages were English, 1 if one parent’s was English, and 2 if neither were English.

**Table S6.** Structure of Likelihood Ratio Tests for Pre-Registered Hypotheses.

| **Covariate Check** | |
| --- | --- |
| m0 | (1\|id) |
| m1 | age + (1\|id) |
| m2 | age + gender + (1\|id) |
| **Profiles of Attention** | |
| m0 | (1\|id) |
| m1 | sig covariates + (1 \|id) |
| m2 | sig covariates + slope + (1+slope\|id) |
| m3 | sig covariates + slope + quadratic + (1+slope+quadratic\|id) |
| *Hypothesis 1: Face Pop-Out* | |
| m4 | sig covariates + slope + quadratic + cubic + (1+slope+quadratic+cubic\|id) |
| m5 | sig covariates + slope + quadratic + cubic + language group + (1+slope+quadratic+cubic) |
| m6 | sig covariates + (slope + quadratic + cubic * language group) + (1+slope+quadratic+cubic) |
| *Hypothesis 2: Dynamic Video* | |
| m4 | sig covariates + slope + quadratic + language group + (1+slope+quadratic) + (1\|scene) |
| m5 | sig covariates + (slope + quadratic * language group) + (1+slope+quadratic) + (1\|scene) |

**Note.** Orthogonal polynomials of stimulus presentation time were computed up to degree three for Face Pop-Out and up to degree two for the Dynamic Video as per Del Bianco et al., 2022. Gender base = female. Language base = monolingual. Stepwise model improvement was tested using Likelihood Ratio Tests.

**Hypothesis 1 – Face Pop-Out: Looking to Faces**

**Table S7.** Infants – Covariate Check for Face Pop-Out: Model Selection Output

| **Model** | **N Parameters** | **AIC** | **BIC** | **Deviance** | **Statistic** | **DF** | ***P*-value** |
| --- | --- | --- | --- | --- | --- | --- | --- |
| Base | 3 | 4807.90 | 4828.60 | 4801.90 | - | - | - |
| + Age | 4 | 4802.50 | 4830.10 | 4794.50 | 7.41 | 1 | .006 ** |
| + Gender | 5 | 4804.20 | 4838.70 | 4794.20 | 0.30 | 1 | .583 |

**Note.** Models compared to base model: *face PLT ~ (1|id)*. ‘+’ marks indicate addition of specified variable as a fixed effect. A *p*-value < 0.05 indicates significant model improvement (i.e., better explanatory power) compared to the base model.

**Table S8.** Infants – Temporal Trends in Face Pop-Out

| **Term** | **Estimate** | **Std Error** | **DF** | ***t*-value** | ***p*-value** |
| --- | --- | --- | --- | --- | --- |
| Intercept | 0.36 | 0.04 | 120.50 | 8.79 | < .001 *** |
| Age | -1.90x10^-4^ | 9.31x10^-5^ | 117.70 | -2.05 | .043 * |
| Slope | -0.16 | 0.06 | 107.50 | -10.76 | < .001 *** |
| Quadratic | 0.06 | 0.01 | 146.20 | 4.04 | < .001 *** |
| Cubic | -0.05 | 0.01 | 118.80 | -3.54 | < .001 *** |

**Note.** Significant effects indicated with *p*-values: * *p* < .05, ** *p* < .01, and *** *p* < .001.

**Table S9.** Infants – Profiles for Face Pop-Out: Model Selection Output

| **Model** | **N Parameters** | **AIC** | **BIC** | **Deviance** | **Statistic** | **DF** | ***P*-value** |
| --- | --- | --- | --- | --- | --- | --- | --- |
| Base | 3 | 4807.90 | 4828.60 | 4801.90 | - | - | - |
| + Age | 4 | 4802.50 | 4830.10 | 4794.50 | 7.41 | 1 | .006 ** |
| + Slope | 5 | 4619.40 | 4653.90 | 4609.40 | 185.03 | 1 | < .001 *** |
| + Quadratic | 11 | 4582.90 | 4658.80 | 4560.90 | 48.53 | 6 | < .001 *** |
| + Cubic | 16 | 4542.30 | 4652.60 | 4510.30 | 50.63 | 5 | < .001 *** |
| + Language Group | 17 | 4543.10 | 4660.30 | 4509.10 | 1.19 | 1 | .275 |
| * Language Group | 20 | 4541.20 | 4679.20 | 4501.20 | 7.87 | 3 | .049 * |

**Note.** Base model: *face PLT ~ (1|id)*. ‘+’ marks indicate addition of specified variable as a fixed effect. ‘*’ indicates addition of an interaction term with 3^rd^ degree polynomials. A *p*-value < 0.05 indicates significant model improvement (i.e., better explanatory power) compared to the base model.

**Table S10.** Bilingual Infants – Face Pop-Out and Within-Group Bilingual Measures: Model Selection Output

| **Model** | **N Parameters** | **AIC** | **BIC** | **Deviance** | **Statistic** | **DF** | ***P*-value** |
| --- | --- | --- | --- | --- | --- | --- | --- |
| Base | 7 | 2191.20 | 2233.70 | 2177.20 | - | - | - |
| *Degree of Bilingualism* | | | | | | | |
| + Degree of Bilingualism | 8 | 2193.20 | 2241.70 | 2177.20 | 0.04 | 1 | .843 |
| * Degree of Bilingualism | 9 | 2194.30 | 2248.90 | 2176.30 | 0.87 | 1 | .352 |
| *Parents’ language Mixing* | | | | | | | |
| + Language Mixing | 8 | 2192.80 | 2241.30 | 2176.80 | 0.42 | 1 | .517 |
| * Language Mixing | 9 | 2194.60 | 2249.20 | 2176.60 | 0.15 | 1 | .702 |

**Note.** Base model: *face PLT ~ slope + age + (1 + slope | id)*. ‘+’ marks indicate addition of specified variable as a fixed effect. ‘*’ indicates addition of an interaction term with slope component. A *p*-value < 0.05 indicates significant model improvement (i.e., better explanatory power) compared to the base model. Degree of bilingualism calculated from language exposure questionnaire (Bosch & Sebastián-Gallés, 1997) as the percentage of exposure to the less dominant language divided by percentage of exposure to the more dominant language (e.g., 40% English / 60% Russian). Language mixing calculated as sum number of language mixing behaviours reported by parent on language mixing scale (Byers-Heinlein et al., 2020).

**Table S11.** Infants – Age Effects on Face Pop-Out: Model Selection Output

| **Model** | **N Parameters** | **AIC** | **BIC** | **Deviance** | **Statistic** | **DF** | ***P*-value** |
| --- | --- | --- | --- | --- | --- | --- | --- |
| Base | 20 | 4541.20 | 4679.20 | 4501.20 | - | - | - |
| * Age | 23 | 4513.70 | 4672.30 | 4467.70 | 33.53 | 3 | < .001 *** |

**Note.** Base model: *face PLT ~ slope + quadratic + cubic + age + (slope + quadratic + cubic) * Language Group + (1 + slope + quadratic + cubic | id)*. ‘+’ marks indicate addition of specified variable as a fixed effect. ‘*’ indicates addition of an interaction term with polynomial components. A *p*-value < 0.05 indicates significant model improvement (i.e., better explanatory power) compared to the base model.

**Table S12.** Infants – Best Fit Model for Face Pop-Out (Hypothesis 1)

| **Term** | **Estimate** | **Std Error** | **DF** | ***t*-value** | ***p*-value** |
| --- | --- | --- | --- | --- | --- |
| Intercept | 0.37 | 0.04 | 116.70 | 8.19 | < .001 *** |
| Slope | -0.16 | 0.06 | 107.50 | -2.76 | .007 ** |
| Quadratic | -0.15 | 0.05 | 580.90 | -3.12 | .002 ** |
| Cubic | 0.21 | 0.05 | 123.50 | 3.98 | < .001 *** |
| Age | -2.48x10^-4^ | 9.61x10^-5^ | 115.50 | -2.57 | .011 * |
| Language Group | 0.03 | 0.02 | 114.00 | 1.30 | .197 |
| Slope x Age | 6.58x10^-5^ | 1.21x10x^-4^ | 97.87 | 0.54 | .588 |
| Quadratic x Age | 4.44x10^-4^ | 1.02x10^-4^ | 516.10 | 4.36 | < .001 *** |
| Cubic x Age | -5.99x10^-4^ | 1.09x10^-4^ | 109.70 | -5.51 | < .001 *** |
| Slope x Language Group | -0.07 | 0.03 | 83.73 | -2.45 | .016 * |
| Quadratic x Language Group | 0.04 | 0.02 | 417.80 | 1.71 | .089 |
| Cubic x Language Group | -0.01 | 0.03 | 88.64 | -0.42 | .676 |

**Note.** ‘x’ indicates interactions. Significant effects indicated with *p*-values: * *p* < .05, ** *p* < .01, and *** *p* < .001.

**Table S13**. Toddlers – Covariate Check for Face Pop-Out: Model Selection Output

| **Model** | **N Parameters** | **AIC** | **BIC** | **Deviance** | **Statistic** | **DF** | ***P*-value** |
| --- | --- | --- | --- | --- | --- | --- | --- |
| Base | 3 | 26711 | 26736 | 26705 |  |  |  |
| + Age | 4 | 26708 | 26743 | 26700 | 4.28 | 1 | .039 * |
| + Gender | 5 | 26708 | 26750 | 26698 | 2.89 | 1 | .089 |

**Note.** Models compared to base model: *face PLT ~ (1|id)*. ‘+’ marks indicate addition of specified variable as a fixed effect. A *p*-value < 0.05 indicates significant model improvement (i.e., better explanatory power) compared to the base model.

**Table S14.** Toddlers – Temporal Trends in Face Pop-Out

| **Term** | **Estimate** | **Std Error** | **DF** | ***t*-value** | ***p*-value** |
| --- | --- | --- | --- | --- | --- |
| Intercept | 0.30 | 4.012e | 551.10 | 7.43 | < .001 *** |
| Age | -6.10x10^-5^ | 6.878x10^-5^ | 549.30 | -0.89 | .376 |
| Slope | -0.21 | 0.01 | 546.40 | -28.76 | < .001 *** |
| Quadratic | 0.13 | 0.01 | 587.80 | 20.32 | < .001 *** |
| Cubic | -0.11 | 0.01 | 605.40 | -18.02 | < .001 *** |

**Note.** Significant effects indicated with *p*-values: * *p* < .05, ** *p* < .01, and *** *p* < .001.

**Table S15.** Toddlers – Profiles for Face Pop-Out: Model Selection Output

| **Model** | **N Parameters** | **AIC** | **BIC** | **Deviance** | **Statistic** | **DF** | ***P*-value** |
| --- | --- | --- | --- | --- | --- | --- | --- |
| Base | 3 | 26711 | 26736 | 26705 | - | - | - |
| + Age | 4 | 26708 | 26743 | 26700 | 4.28 | 1 | .039 * |
| + Slope | 7 | 24832 | 24892 | 24818 | 1882.79 | 3 | < .001 *** |
| + Quadratic | 11 | 24056 | 24150 | 24034 | 783.83 | 4 | < .001 *** |
| + Cubic | 16 | 23503 | 23640 | 23471 | 562.76 | 5 | < .001 *** |
| *Categorical group predictor* | | | | | | | |
| + Language Group | 17 | 23505 | 23651 | 23471 | 0.01 | 1 | .931 |
| * Language Group | 20 | 23508 | 23680 | 23468 | 2.90 | 3 | .407 |
| *Numeric group predictor* | | | | | | | |
| + Language Group | 17 | 23505 | 23651 | 23471 | 0.21 | 1 | .650 |
| * Language Group | 20 | 23509 | 23681 | 23469 | 1.56 | 3 | .668 |

**Note.** Models compared to base model: *face PLT ~ (1|id)*. ‘+’ marks indicate addition of specified variable as a fixed effect. ‘*’ indicates addition of an interaction term with 3^rd^ degree polynomials. A *p*-value < 0.05 indicates significant model improvement (i.e., better explanatory power) compared to the base model. Categorical group predictor calculated as monolingual if toddlers’ mothers’ first language was English, bilingual if it was not English. Numeric group predictor calculated as 0 if both parents’ first languages were English, 1 if one parent’s was English, and 2 if neither were English.

**Table S16.** Toddlers – Age Effects on Face Pop-Out: Model Selection Output

| **Model** | **N Parameters** | **AIC** | **BIC** | **Deviance** | **Statistic** | **DF** | ***P*-value** |
| --- | --- | --- | --- | --- | --- | --- | --- |
| Base | 16 | 23503 | 23640 | 23471 | - | - | - |
| * Age | 19 | 23485 | 23648 | 23447 | 24.43 | 3 | < .001 *** |

**Note.** Models compared to base model: *face PLT ~ (slope + quadratic + cubic) + (1 + slope + quadratic + cubic | id)*. ‘+’ marks indicate addition of specified variable as a fixed effect. ‘*’ indicates addition of an interaction term with 3^rd^ degree polynomials. A *p*-value < 0.05 indicates significant model improvement (i.e., better explanatory power) compared to the base model.

**Table S17.** Toddlers – Best Fit Model for Face Pop-Out (Hypothesis 1)

| **Term** | **Estimate** | **Std Error** | **DF** | ***t*-value** | ***p*-value** |
| --- | --- | --- | --- | --- | --- |
| Intercept | 0.35 | 0.04 | 549.90 | 8.34 | < .001 *** |
| Slope | -0.47 | 0.06 | 534.40 | -7.95 | < .001 *** |
| Quadratic | 0.27 | 0.05 | 575.60 | 5.23 | < .001 *** |
| Cubic | -0.23 | 0.05 | 595.70 | -4.65 | < .001 *** |
| Age | -1.47x10^-4^ | 7.17x10^-5^ | 549.60 | -2.06 | .040 * |
| Slope x Age | 4.40x10^-4^ | 1.01x10^-4^ | 533.30 | 4.35 | < .001 *** |
| Quadratic x Age | -2.44x10^-4^ | 8.98x10^-5^ | 574.20 | -2.71 | .007 ** |
| Cubic x Age | 2.02x10^-4^ | 8.32x10^-5^ | 594.00 | 2.42 | .016 * |

**Note.** ‘x’ indicates interactions. Significant effects indicated with *p*-values: * for *p* < .05, ** for *p* < .01, and *** for *p* < .001.

**Hypothesis 2 – Dynamic Video: Looking to Mouths**

**Table S18.** Infants – Covariate Check for Dynamic Video: Model Selection Output

| **Model** | **N Parameters** | **AIC** | **BIC** | **Deviance** | **Statistic** | **DF** | ***P*-value** |
| --- | --- | --- | --- | --- | --- | --- | --- |
| Base | 3 | 2536.80 | 2558.00 | 2530.80 | - | - | - |
| + Age | 4 | 2520.80 | 2549.10 | 2512.80 | 18.00 | 1 | < .001 *** |
| + Gender | 5 | 2517.80 | 2553.10 | 2507.80 | 5.03 | 1 | .025 * |

**Note.** Base model: *mouth PLT ~ (1 | id)*. ‘+’ marks indicate addition of specified variable as a fixed effect. A *p*-value < 0.05 indicates significant model improvement (i.e., better explanatory power) compared to the base model.

**Table S19.** Infants – Temporal Trends in Dynamic Video

| **Term** | **Estimate** | **Std Error** | **DF** | ***t*-value** | ***p*-value** |
| --- | --- | --- | --- | --- | --- |
| Intercept | 0.02 | 4.051e | 121.10 | 0.46 | .646 |
| Age | 4.20x10^-4^ | 9.112e | 121.60 | 4.61 | < .001 *** |
| Gender | -0.05 | 2.361e | 121.40 | -2.23 | .028 * |
| Slope | -0.03 | 7.499e | 196.30 | -4.07 | < .001 *** |
| Quadratic | 3.63x10^-3^ | 7.370e | 240.40 | 0.49 | .623 |

**Note.** Significant effects indicated with *p*-values: * *p* < .05, ** *p* < .01, and *** *p* < .001.

**Table S20.** Infants – Profiles for Dynamic Video: Model Selection Output

| **Model** | **N Parameters** | **AIC** | **BIC** | **Deviance** | **Statistic** | **DF** | ***P*-value** |
| --- | --- | --- | --- | --- | --- | --- | --- |
| Base | 3 | 2536.80 | 2558.00 | 2530.80 | - | - | - |
| + Age | 4 | 2520.80 | 2549.10 | 2512.80 | 18.00 | 1 | < .001 *** |
| + Gender | 5 | 2517.80 | 2553.10 | 2507.80 | 5.03 | 1 | .025 * |
| + Slope | 6 | 2500.40 | 2542.80 | 2488.40 | 19.36 | 1 | < .001 *** |
| + Quadratic | 12 | 2507.40 | 2592.20 | 2483.40 | 5.07 | 6 | .534 |
| + Language Group | 13 | 2507.20 | 2599.10 | 2481.20 | 2.16 | 1 | .141 |
| * Language Group | 15 | 2505.30 | 2611.30 | 2475.30 | 5.92 | 2 | .052 |

**Note.** Base model: *mouth PLT ~ (1 | id)*. ‘+’ marks indicate addition of specified variable as a fixed effect. ‘*’ indicates addition of an interaction term with 2^nd^ degree polynomials. A *p*-value < 0.05 indicates significant model improvement (i.e., better explanatory power) compared to the base model.

**Table S21.** Infants – Age Effects on Dynamic Video: Model Selection Output

| **Model** | **N Parameters** | **AIC** | **BIC** | **Deviance** | **Statistic** | **DF** | ***P*-value** |
| --- | --- | --- | --- | --- | --- | --- | --- |
| Base | 8 | 2504.10 | 2560.60 | 2488.10 | - | - | - |
| * Age | 9 | 2503.20 | 2566.80 | 2485.20 | 2.88 | 1 | .090 |

**Note.** Base model: *mouth PLT ~ age + gender + slope + (1 + slope | id)*. ‘*’ indicates addition of an interaction term with slope and language group term. A *p*-value < 0.05 indicates significant model improvement (i.e., better explanatory power) compared to the base model.

**Table S22.** Infants – Language Group Effects on Dynamic Video: Model Selection Output

| **Model** | **N Parameters** | **AIC** | **BIC** | **Deviance** | **Statistic** | **DF** | ***P*-value** |
| --- | --- | --- | --- | --- | --- | --- | --- |
| Base | 8 | 2504.10 | 2560.60 | 2488.10 | - | - | - |
| + Language Group | 9 | 2504.00 | 2567.60 | 2486.00 | 2.10 | 1 | .148 |
| * Language Group | 10 | 2501.10 | 2571.80 | 2481.10 | 4.87 | 1 | .027 * |

**Note.** Base model: *mouth PLT ~ age + gender + slope + (1 + slope | id)*. ‘+’ marks indicate addition of specified variable as a fixed effect. ‘*’ indicates addition of an interaction term with slope component. A *p*-value < 0.05 indicates significant model improvement (i.e., better explanatory power) compared to the base model.

**Table S23.** Infants – Best Fit Model for Dynamic Video (Hypothesis 2)

| **Term** | **Estimate** | **Std Error** | **DF** | ***t*-value** | ***p*-value** |
| --- | --- | --- | --- | --- | --- |
| Intercept | -1.71x10^-3^ | 0.04 | 120.20 | -0.04 | .968 |
| Age | 4.32x10^-4^ | 9.11x10^-5^ | 120.80 | 4.74 | < .001 *** |
| Gender | -0.05 | 0.02 | 120.70 | -2.27 | .025 * |
| Slope | -0.02 | 0.01 | 121.30 | -1.72 | .089 |
| Language Group | 0.04 | 0.02 | 120.90 | 1.51 | .134 |
| Slope x Language Group | -0.03 | 0.01 | 121.20 | -2.22 | .029 * |

**Note.** ‘x’ indicates interactions. Significant effects indicated with *p*-values: * for *p* < .05, ** for *p* < .01, and *** for *p* < .001.

**Table S24.** Bilingual Infants – Dynamic Video and Within-Group Bilingual Measures: Model Selection Output

| **Model** | **N Parameters** | **AIC** | **BIC** | **Deviance** | **Statistic** | **DF** | ***P*-value** |
| --- | --- | --- | --- | --- | --- | --- | --- |
| Base | 8 | 1356.60 | 1406.20 | 1340.60 | - | - | - |
| *Degree of Bilingualism* | | | | | | | |
| + Degree of Bilingualism | 9 | 1358.50 | 1414.20 | 1340.50 | 0.10 | 1 | .754 |
| * Degree of Bilingualism | 10 | 1357.30 | 1419.30 | 1337.30 | 3.17 | 1 | .075 |
| *Parents’ language Mixing* | | | | | | | |
| + Language Mixing | 9 | 1358.60 | 1414.30 | 1340.60 | 0.00 | 1 | .996 |
| * Language Mixing | 10 | 1359.70 | 1421.60 | 1339.70 | 0.94 | 1 | .332 |
| *Percentage Exposure to English* | | | | | | | |
| + Percentage English Exposure | 9 | 1358.60 | 1414.30 | 1340.60 | 0.00 | 1 | .984 |
| * Percentage English Exposure | 10 | 1369.60 | 1422.50 | 1340.60 | 0.03 | 1 | .860 |

**Note.** Base model: *mouth PLT ~ age + gender + slope + (1 + slope | id)*. ‘+’ marks indicate addition of specified variable as a fixed effect. ‘*’ indicates addition of an interaction term with slope component. A *p*-value < 0.05 indicates significant model improvement (i.e., better explanatory power) compared to the base model. Degree of bilingualism calculated from language exposure questionnaire (Bosch & Sebastián-Gallés, 1997) as the percentage of exposure to the less dominant language divided by percentage of exposure to the more dominant language (e.g., 40% English / 60% Russian). Language mixing calculated as sum number of language mixing behaviours reported by parent on language mixing scale (Byers-Heinlein et al., 2020). Percentage of English exposure calculated from language exposure questionnaire (Bosch & Sebastián-Gallés, 1997) as children’s overall percentage of exposure to English.

**Table S25.** Toddlers – Covariate Check for Dynamic Video: Model Selection Output

| **Model** | **N Parameters** | **AIC** | **BIC** | **Deviance** | **Statistic** | **DF** | ***P*-value** |
| --- | --- | --- | --- | --- | --- | --- | --- |
| Base | 3 | 16780 | 16805 | - | - | - | - |
| + Age | 4 | 16782 | 16816 | 16774 | 0.00 | 1 | .976 |
| + Gender | 5 | 16783 | 16825 | 16773 | 1.20 | 1 | .273 |

**Note.** Models compared to base model: *mouth PLT ~ (1|id)*. ‘+’ marks indicate addition of specified variable as a fixed effect. A *p*-value < 0.05 indicates significant model improvement (i.e., better explanatory power) compared to the base model. Categorical group predictor calculated as monolingual if infants’ mothers’ first language was English, bilingual if it was not English. Numeric group predictor calculated as 0 if both parents’ first languages were English, 1 if one parent’s was English, and 2 if neither were English.

**Table S26.** Toddlers – Temporal Trends in Dynamic Video

| **Term** | **Estimate** | **Std Error** | **DF** | ***t*-value** | ***p*-value** |
| --- | --- | --- | --- | --- | --- |
| Intercept | 0.19 | 0.01 | 541.76 | 31.00 | < .001 *** |
| Slope | 0.08 | 0.01 | 650.33 | 14.83 | < .001 *** |
| Quadratic | -0.11 | 0.01 | 718.45 | -20.67 | < .001 *** |

**Note.** Significant effects indicated with *p*-values: * *p* < .05, ** *p* < .01, and *** *p* < .001.

**Table S27.** Toddlers – Profiles for Dynamic Video: Model Selection Output

| **Model** | **N Parameters** | **AIC** | **BIC** | **Deviance** | **Statistic** | **DF** | ***P*-value** |
| --- | --- | --- | --- | --- | --- | --- | --- |
| Base | 3 | 16780 | 16805 | 16774 | - | - | - |
| + Slope | 4 | 16061 | 16111 | 16049 | 725.38 | 3 | < .001 *** |
| + Quadratic | 10 | 14965 | 15048 | 14945 | 1103.98 | 4 | < .001 *** |
| *Categorical group predictor* | | | | | | | |
| + Language Group | 11 | 14962 | 15053 | 14940 | 5.24 | 1 | .022 * |
| * Language Group | 13 | 14959 | 15068 | 14933 | 6.50 | 2 | .039 * |
| *Numeric group predictor* | | | | | | | |
| + Language Group | 11 | 14964 | 15056 | 14942 | 2.99 | 1 | .089 |
| * Language Group | 13 | 14962 | 15071 | 14936 | 5.70 | 2 | .058 |

**Note.** Models compared to base model: *mouth PLT ~ (1|id).* ‘+’ marks indicate addition of specified variable as a fixed effect. ‘*’ indicates addition of an interaction term with 2^nd^ degree polynomials. A *p*-value < 0.05 indicates significant model improvement (i.e., better explanatory power) compared to the base model. Categorical group predictor calculated as monolingual if toddlers’ mothers’ first language was English, bilingual if it was not English. Numeric group predictor calculated as 0 if both parents’ first languages were English, 1 if one parent’s was English, and 2 if neither were English.

**Table S28.** Toddlers – Best Fit Model for Dynamic Video (Hypothesis 2)

| **Term** | **Estimate** | **Std Error** | **DF** | ***t*-value** | ***p*-value** |
| --- | --- | --- | --- | --- | --- |
| Intercept | 0.17 | 0.01 | 538.40 | 22.55 | < .001 *** |
| Slope | 0.07 | 0.01 | 642.44 | 10.30 | < .001 *** |
| Quadratic | -0.10 | 0.01 | 710.64 | -15.26 | < .001 *** |
| Language Group | 0.04 | 0.01 | 541.23 | 3.39 | .001 ** |
| Slope x Language Group | 0.03 | 0.01 | 649.46 | 2.36 | .019 * |
| Quadratic x Language Group | -0.02 | 0.01 | 722.27 | -1.73 | .085 |

**Note.** ‘x’ indicates interactions. Significant effects indicated with *p*-values: * for *p* < .05, ** for *p* < .01, and *** for *p* < .001.

**Table S29.** Developmental Effects in Face Pop-Out (All Children): Model Selection Output

| **Model** | **N Parameters** | **AIC** | **BIC** | **Deviance** | **Statistic** | **DF** | ***P*-value** |
| --- | --- | --- | --- | --- | --- | --- | --- |
| Base | 3 | 31517 | 31543 | 31511 | - | - | - |
| + Slope | 6 | 29442 | 29494 | 29430 | 2081.33 | 3 | < .001 *** |
| + Quadratic | 10 | 28662 | 28750 | 28642 | 787.11 | 4 | < .001 *** |
| + Cubic | 15 | 28073 | 28204 | 28043 | 599.46 | 5 | < .001 *** |
| + Age | 16 | 28065 | 28205 | 28033 | 9.66 | 1 | .002 ** |
| * Age | 19 | 28052 | 28219 | 28014 | 18.86 | 3 | < .001 *** |

**Note.** Both samples of infants and toddlers collapsed to test one developmental model across both groups (*N* = 876). Age calculated as a continuous predictor in days. Base model: *face PLT ~ (1|id)*. ‘+’ marks indicate addition of specified variable as a fixed effect. ‘*’ indicates addition of an interaction term with 3^rd^ degree polynomials. A *p*-value < 0.05 indicates significant model improvement (i.e., better explanatory power) compared to the base model.

**Table S30.** Developmental Effects in Face Pop-Out (All Children): Best Fit Model

| **Term** | **Estimate** | **Std Error** | **DF** | ***t*-value** | ***p*-value** |
| --- | --- | --- | --- | --- | --- |
| Intercept | 0.35 | 0.03 | 690.50 | 13.68 | < .001 *** |
| Slope | -0.27 | 0.04 | 773.80 | -7.05 | < .001 *** |
| Quadratic | 0.03 | 0.03 | 872.20 | 0.81 | .417 |
| Cubic | -4.93x10^-3^ | 0.03 | 893.30 | -0.15 | .877 |
| Age | -1.56x10^-4^ | 4.59x10^-5^ | 687.90 | -3.41 | < .001 *** |
| Slope x Age | 1.07x10^-4^ | 6.68x10^-5^ | 759.20 | 1.60 | .109 |
| Quadratic x Age | 1.67x10^-4^ | 5.98x10^-5^ | 852.20 | 2.80 | .005 ** |
| Cubic x Age | -1.72x10^-4^ | 5.67x10^-5^ | 870.80 | -3.04 | .002 ** |

**Note.** Both samples of infants and toddlers collapsed to test one developmental model across both groups (*N* = 876). Age calculated as a continuous predictor in days. ‘x’ indicates interactions. Significant effects indicated with *p*-values: * for *p* < .05, ** for *p* < .01, and *** for *p* < .001.

**Table S31.** Developmental Effects in Dynamic Video (All Children): Model Selection Output

| **Model** | **N Parameters** | **AIC** | **BIC** | **Deviance** | **Statistic** | **DF** | ***P*-value** |
| --- | --- | --- | --- | --- | --- | --- | --- |
| Base | 3 | 19492 | 19518 | 19486 | - | - | - |
| + Slope | 4 | 19233 | 19267 | 19225 | 261.16 | 1 | < .001 *** |
| + Quadratic | 10 | 17953 | 18039 | 17933 | 1291.75 | 6 | < .001 *** |
| + Age | 11 | 17949 | 18043 | 17927 | 6.65 | 1 | .010 * |
| * Age | 13 | 17885 | 17997 | 17859 | 67.32 | 2 | < .001 *** |

**Note.** Both samples of infants and toddlers collapsed to test one developmental model across both groups (*N* = 876). Age calculated as a continuous predictor in days. Base model: *mouth PLT ~ (1|id)*. ‘+’ marks indicate addition of specified variable as a fixed effect. ‘*’ indicates addition of an interaction term with 2^nd^ degree polynomials. A *p*-value < 0.05 indicates significant model improvement (i.e., better explanatory power) compared to the base model.

**Table S32.** Developmental Effects in Dynamic Video (All Children): Best Fit Model

| **Term** | **Estimate** | **Std Error** | **DF** | ***t*-value** | ***p*-value** |
| --- | --- | --- | --- | --- | --- |
| Intercept | 0.09 | 0.03 | 648.40 | 3.27 | .001 ** |
| Slope | -0.09 | 0.02 | 584.30 | -3.99 | < .001 *** |
| Quadratic | 0.07 | 0.02 | 600.20 | 3.00 | < .001 *** |
| Age | 1.70x10^-4^ | 4.96x10^-5^ | 650.90 | 3.42 | < .001 *** |
| Slope x Age | 2.70x10^-4^ | 2.70x10^-5^ | 598.90 | 6.63 | < .001 *** |
| Quadratic x Age | -2.82x10^-4^ | 3.99x10^-5^ | 615.00 | -7.06 | < .001 *** |

**Note.** Both samples of infants and toddlers collapsed to test one developmental model across both groups (*N* = 876). Age calculated as a continuous predictor in days. ‘x’ indicates interactions. Significant effects indicated with *p*-values: * for *p* < .05, ** for *p* < .01, and *** for *p* < .001.

**Table S33.** Infants – Profiles for Eyes in Dynamic Video: Model Selection Output

| **Model** | **N Parameters** | **AIC** | **BIC** | **Deviance** | **Statistic** | **DF** | ***P*-value** |
| --- | --- | --- | --- | --- | --- | --- | --- |
| Base | 4 | 4051.70 | 4080.00 | 4043.70 | - | - | - |
| + Slope | 5 | 4044.30 | 4079.70 | 4034.30 | 9.43 | 1 | .002 ** |
| + Quadratic | 11 | 4052.90 | 4130.70 | 4030.90 | 3.40 | 6 | .758 |
| + Language Group | 12 | 4054.90 | 4139.70 | 4030.90 | 0.03 | 1 | .862 |
| * Language Group | 14 | 4056.70 | 4155.70 | 4028.70 | 2.16 | 2 | .340 |

**Note.** An average profile model revealed a main effect of age which was therefore added to the based model. Base model: *eye PLT ~ age + (1|id)*. ‘+’ marks indicate addition of specified variable as a fixed effect. ‘*’ indicates addition of an interaction term with 2^nd^ degree polynomials. A *p*-value < 0.05 indicates significant model improvement (i.e., better explanatory power) compared to the base model.

**Table S34.** Infants – Profiles for Eyes in Dynamic Video: Best Fit Model

| **Term** | **Estimate** | **Std Error** | **DF** | ***t*-value** | ***p*-value** |
| --- | --- | --- | --- | --- | --- |
| Intercept | 0.27 | 0.03 | 123.30 | 7.91 | < .001 *** |
| Age | -1.65x10^-4^ | 8.06x10^-5^ | 123.90 | -2.04 | .043 * |
| Slope | 0.02 | 0.01 | 857.60 | 3.07 | .002 ** |

**Note.** Significant effects indicated with *p*-values: * for *p* < .05, ** for *p* < .01, and *** for *p* < .001.

**Table S35.** Toddlers – Profiles for Eyes in Dynamic Video: Model Selection Output

| **Model** | **N Parameters** | **AIC** | **BIC** | **Deviance** | **Statistic** | **DF** | ***P*-value** |
| --- | --- | --- | --- | --- | --- | --- | --- |
| Base | 3 | 13536 | 13561 | 13530 | - | - | - |
| + Slope | 6 | 12683 | 12733 | 12671 | 858.64 | 3 | < .001 *** |
| + Quadratic | 10 | 12414 | 12498 | 12394 | 276.38 | 4 | < .001 *** |
| *Categorical group predictor* | | | | | | | |
| + Language Group | 11 | 12414 | 12507 | 12392 | 2.04 | 1 | .154 |
| * Language Group | 13 | 12416 | 12525 | 12390 | 2.65 | 2 | .266 |
| *Numeric group predictor* | | | | | | | |
| + Language Group | 11 | 12415 | 12507 | 12393 | 1.62 | 1 | .203 |
| * Language Group | 13 | 12417 | 12526 | 12391 | 1.52 | 2 | .468 |

**Note.** Average profile models revealed no main effects of age nor gender. Base model: *eye PLT ~ (1|id)*. ‘+’ marks indicate addition of specified variable as a fixed effect. ‘*’ indicates addition of an interaction term with 2^nd^ degree polynomials. Categorical group predictor calculated as monolingual if toddlers’ mothers’ first language was English, bilingual if it was not English. Numeric group predictor calculated as 0 if both parents’ first languages were English, 1 if one parent’s was English, and 2 if neither were English. A *p*-value < 0.05 indicates significant model improvement (i.e., better explanatory power) compared to the base model.

**Table S36.** Toddlers – Profiles for Eyes in Dynamic Video: Best Fit Model

| **Term** | **Estimate** | **Std Error** | **DF** | ***t*-value** | ***p*-value** |
| --- | --- | --- | --- | --- | --- |
| Intercept | 0.18 | 0.01 | 565.22 | 34.58 | < .001 *** |
| Slope | 0.09 | 0.00 | 699.66 | 18.93 | < .001 *** |
| Quadratic | -0.04 | 0.00 | 936.30 | - 9.80 | < .001 *** |

**Note.** Significant effects indicated with *p*-values: * for *p* < .05, ** for *p* < .01, and *** for *p* < .001.

**Table S37.** Infants – Profiles for Faces in Dynamic Video: Model Selection Output

| **Model** | **N Parameters** | **AIC** | **BIC** | **Deviance** | **Statistic** | **DF** | ***P*-value** |
| --- | --- | --- | --- | --- | --- | --- | --- |
| Base | 4 | 8100.20 | 8128.50 | 8092.20 | - | - | - |
| + Slope | 5 | 8071.60 | 8107.00 | 8061.60 | 30.60 | 1 | < .001 *** |
| + Quadratic | 11 | 8079.70 | 8157.50 | 8057.70 | 3.87 | 6 | .694 |
| + Language Group | 12 | 8080.20 | 8165.10 | 8056.20 | 1.52 | 1 | .218 |
| * Language Group | 14 | 8084.00 | 8183.00 | 8056.00 | 0.21 | 2 | .900 |

**Note.** An average profile model revealed a main effect of gender which was therefore added to the based model. Base model: *eye PLT ~ gender + (1|id)*. ‘+’ marks indicate addition of specified variable as a fixed effect. ‘*’ indicates addition of an interaction term with 2^nd^ degree polynomials. A *p*-value < 0.05 indicates significant model improvement (i.e., better explanatory power) compared to the base model.

**Table S38.** Infants – Profiles for Faces in Dynamic Video: Best Fit Model

| **Term** | **Estimate** | **Std Error** | **DF** | ***t*-value** | ***p*-value** |
| --- | --- | --- | --- | --- | --- |
| Intercept | 0.67 | 0.02 | 121.70 | 29.31 | < .001 *** |
| Gender | -0.06 | 0.03 | 122.20 | -2.12 | .036 * |
| Slope | -0.05 | 0.01 | 8574.00 | -5.54 | < .001 *** |

**Note.** Significant effects indicated with *p*-values: * for *p* < .05, ** for *p* < .01, and *** for *p* < .001.

**Table S39.** Toddlers – Profiles for Faces in Dynamic Video: Model Selection Output

| **Model** | **N Parameters** | **AIC** | **BIC** | **Deviance** | **Statistic** | **DF** | ***P*-value** |
| --- | --- | --- | --- | --- | --- | --- | --- |
| Base | 3 | 29722 | 29747 | 29716 | - | - | - |
| + Slope | 6 | 27093 | 27143 | 27081 | 2635.13 | 3 | < .001 *** |
| + Quadratic | 10 | 24545 | 24629 | 24525 | 2555.67 | 4 | < .001 *** |
| *Categorical group predictor* | | | | | | | |
| + Language Group | 11 | 24547 | 24639 | 24525 | 0.08 | 1 | .776 |
| * Language Group | 13 | 24547 | 24656 | 24521 | 4.16 | 2 | .125 |
| *Numeric group predictor* | | | | | | | |
| + Language Group | 11 | 24547 | 24639 | 24525 | 0.14 | 1 | .706 |
| * Language Group | 13 | 24549 | 24658 | 24523 | 1.74 | 2 | .419 |

**Note.** Average profile models revealed no main effects of age nor gender. Base model: *mouth PLT ~ (1|id)*. ‘+’ marks indicate addition of specified variable as a fixed effect. ‘*’ indicates addition of an interaction term with 2^nd^ degree polynomials. Categorical group predictor calculated as monolingual if toddlers’ mothers’ first language was English, bilingual if it was not English. Numeric group predictor calculated as 0 if both parents’ first languages were English, 1 if one parent’s was English, and 2 if neither were English. A *p*-value < 0.05 indicates significant model improvement (i.e., better explanatory power) compared to the base model.

**Table S40.** Toddlers – Profiles for Faces in Dynamic Video: Best Fit Model

| **Term** | **Estimate** | **Std Error** | **DF** | ***t*-value** | ***p*-value** |
| --- | --- | --- | --- | --- | --- |
| Intercept | 0.61 | 0.01 | 554.79 | 74.27 | < .001 *** |
| Slope | 0.23 | 0.01 | 522.55 | 38.47 | < .001 *** |
| Quadratic | -0.21 | 0.01 | 912.40 | -37.47 | < .001 *** |

**Note.** Significant effects indicated with *p*-values: * for *p* < .05, ** for *p* < .01, and *** for *p* < .001.

**Table S41.** Post-hoc categorisation of non-English language distance from English.

|  | **Non-English Languages** |
| --- | --- |
| **Close non-English language pairs** | Affrikans [Afrikaans], Albanian, Bangla [Bengali], Bulgarian, Catalan, Croatian, Czech, Danish, Dari, Dutch, Farsi [Persian], Flemish [Dutch], French, German, Greek, Hindi, Irish, Italian, Konkani, Kurdish, Lithuanian, Marathi, Nepali, Norwegian, Polish, Portuguese, Punjabi [Panjabi], Romanian, Russian, Sinhala, Slovak, Spanish, Swedish, Swiss German [German], Ukranian, Urdu, Welsh |
| **Far non-English language pairs** | Amharic, Arabic, Beni, Benin, Bilen, Burmese, Cantonese, Chinese (unspecified) [Mandarin], Creole, Estonian, Ethiopian, Filipino (Bisaya) [Tagalog], Filipino (unspecified) [Tagalog], Finnish, Ga, Georgian, Hausa, Hebrew, Hungarian, Ijaw, Indonesian, Itsekiri, Japanese, Kiro, Korean, Luganda, Lunyoro, Malay, Malayalam, Mandarin, Mamprusi [Mampruli], Mandinka, Siswati [Swati], Somali, Swahili, Tagalog, Tamil, Temne, Thai, Tigrinea [Tigrinya], Turkish, Turkmen, Twi [Akan], Urhobo, Uzbek, Vietnamese, Wolouf [Wolof], Yoruba |

**Note.** Non-English language distance to English was categorised as “close” when listed as within the Indo-European language family according to the WALS online tool (Dryer & Haspelmath, 2013). Languages listed as belonging to a different language family (e.g., Amharic), not listed on the tool (i.e., Bilen, Itsekiri, Ga, Lunyoro), or not specifically indicated by parent (i.e., Benin, Ethiopian, Ijaw, Kiro) were categorised as “far”. Square brackets indicate the entry used for search in place of the parent-listed language.

**Table S42.** Bilingual Infants – Language Distance and Profiles for Face Pop-Out: Model Selection Output

| **Model** | **N Parameters** | **AIC** | **BIC** | **Deviance** | **Statistic** | **DF** | ***P*-value** |
| --- | --- | --- | --- | --- | --- | --- | --- |
| Base | 15 | 2343.80 | 2435.90 | 2313.80 | - | - | - |
| + Language Distance | 16 | 2344.20 | 2442.40 | 2312.20 | 1.57 | 1 | .210 |
| * Language Distance | 19 | 2344.90 | 2461.50 | 2306.90 | 5.36 | 3 | .147 |

**Note.** Base model: *face PLT ~ slope + quadratic + cubic + (1+slope+quadratic+cubic|id)*. ‘+’ marks indicate addition of specified variable as a fixed effect. ‘*’ indicates addition of an interaction term with 3^rd^ degree polynomials. A *p*-value < 0.05 indicates significant model improvement (i.e., better explanatory power) compared to the base model.

**Table S43.** Toddlers with Bilingual Mothers – Language Distance and Profiles for Face Pop-Out: Model Selection Output

| **Model** | **N Parameters** | **AIC** | **BIC** | **Deviance** | **Statistic** | **DF** | ***P*-value** |
| --- | --- | --- | --- | --- | --- | --- | --- |
| Base | 15 | 9161.70 | 9276.00 | 9131.70 | - | - | - |
| + Language Distance | 16 | 9163.60 | 9285.60 | 9131.60 | 0.11 | 1 | .738 |
| * Language Distance | 19 | 9165.50 | 9310.30 | 9127.50 | 4.12 | 3 | .249 |

**Note.** Base model: *face PLT ~ slope + quadratic + cubic + (1+slope+quadratic+cubic|id)*. ‘+’ marks indicate addition of specified variable as a fixed effect. ‘*’ indicates addition of an interaction term with 3^rd^ degree polynomials. A *p*-value < 0.05 indicates significant model improvement (i.e., better explanatory power) compared to the base model.

**Table S44.** Bilingual Infants – Language Distance and Profiles for Dynamic Video: Model Selection Output

| **Model** | **N Parameters** | **AIC** | **BIC** | **Deviance** | **Statistic** | **DF** | ***P*-value** |
| --- | --- | --- | --- | --- | --- | --- | --- |
| Base | 10 | 1580.40 | 1643.40 | 1560.40 | - | - | - |
| + Language Distance | 11 | 1582.20 | 1651.40 | 1560.20 | .23 | 1 | .630 |
| * Language Distance | 13 | 1584.80 | 1666.70 | 1558.80 | 1.35 | 2 | .508 |

**Note.** Base model: *face PLT ~ slope + quadratic + (1+slope+quadratic|id)*. ‘+’ marks indicate addition of specified variable as a fixed effect. ‘*’ indicates addition of an interaction term with 2^nd^ degree polynomials. A *p*-value < 0.05 indicates significant model improvement (i.e., better explanatory power) compared to the base model.

**Table S45.** Toddlers with Bilingual Mothers – Language Distance and Profiles for Dynamic Video: Model Selection Output

| **Model** | **N Parameters** | **AIC** | **BIC** | **Deviance** | **Statistic** | **DF** | ***P*-value** |
| --- | --- | --- | --- | --- | --- | --- | --- |
| Base | 10 | 7104.30 | 7178.00 | 7084.30 | - | - | - |
| + Language Distance | 11 | 7102.80 | 7183.90 | 7080.80 | 3.48 | 1 | .062 |
| * Language Distance | 13 | 7106.10 | 7201.90 | 7080.10 | 0.71 | 2 | .702 |

**Note.** Base model: *face PLT ~ slope + quadratic + (1+slope+quadratic|id)*. ‘+’ marks indicate addition of specified variable as a fixed effect. ‘*’ indicates addition of an interaction term with 2^nd^ degree polynomials. A *p*-value < 0.05 indicates significant model improvement (i.e., better explanatory power) compared to the base model.
